# Bioarchaeological evidence of one of the earliest Islamic burials in the Levant

**DOI:** 10.1038/s42003-022-03508-4

**Published:** 2022-06-07

**Authors:** Megha Srigyan, Héctor Bolívar, Irene Ureña, Jonathan Santana, Andrew Petersen, Eneko Iriarte, Emrah Kırdök, Nora Bergfeldt, Alice Mora, Mattias Jakobsson, Khaled Abdo, Frank Braemer, Colin Smith, Juan José Ibañez, Anders Götherström, Torsten Günther, Cristina Valdiosera

**Affiliations:** 1 Human Evolution, Department of Organismal Biology, Uppsala University, Uppsala, Sweden; 2 Centre for Palaeogenetics, 10691 Stockholm, Sweden; 3 Instituto del Patrimonio Cultural de España, 28040 Madrid, Spain; 4 Department of Historical Sciences, Universidad de Las Palmas de Gran Canaria, Las Palmas de G.C., E35001 Spain; 5 University of Wales Trinity Saint David, Lampeter, UK; 6 Laboratorio de Evolución Humana, Departamento de Historia, Geografía y Comunicación, Universidad de Burgos, 09001 Burgos, Spain; 7 Department of Biotechnology, Mersin University, 33343 Mersin, Turkey; 8 Dept. Archaeology and History, La Trobe University, Melbourne, VIC 3086 Australia; 9 General Directorate of Antiquities and Museums, Damascus, Syrian Arab Republic; 10 Université Côte d’Azur, CNRS, Culture et Environment, Préhistoire Antiquité Moyen Age, Nice, France; 11 Archaeology of Social Dynamics, Milà i Fontanals Institution, Spanish National Research Council (CSIC), Barcelona, Spain; 12 Present Address: Department of Ecology and Evolutionary Biology, University of California Santa Cruz, Santa Cruz, CA 95064 USA

**Keywords:** Archaeology, Population genetics, Population genetics, Anthropology

## Abstract

The Middle East plays a central role in human history harbouring a vast diversity of ethnic, cultural and religious groups. However, much remains to be understood about past and present genomic diversity in this region. Here we present a multidisciplinary bioarchaeological analysis of two individuals dated to the late 7th and early 8th centuries, the Umayyad Era, from Tell Qarassa, an open-air site in modern-day Syria. Radiocarbon dates and burial type are consistent with one of the earliest Islamic Arab burials in the Levant. Interestingly, we found genomic similarity to a genotyped group of modern-day Bedouins and Saudi rather than to most neighbouring Levantine groups. This study represents the genomic analysis of a secondary use site with characteristics consistent with an early Islamic burial in the Levant. We discuss our findings and possible historic scenarios in the light of forces such as genetic drift and their possible interaction with religious and cultural processes (including diet and subsistence practices).

## Abstract (web summary)

Ancient genomic and archaeological data combine to identify a surprisingly early Islamic burial in modern day Syria.

## Introduction

The Middle East has an unparalleled place in human history. From the out-of-Africa movements of modern humans and admixture with Neandertals, to the spread of agriculture and emergence of civilisations, it has been at the crossroads of genetic as well as cultural history for millennia. While archaeological and historical sources provide instrumental insights into socio-cultural aspects, ancient DNA (aDNA) enables the recovery of past genetic information, filling crucial gaps in our understanding of population history. A number of aDNA studies from different time periods in the Middle East have provided a general overview of the genetic history in this region. These include descriptions of the earliest local farming groups from the Neolithic^1^ and their expansions into Europe^2,3^ as well as genetic differentiation among contemporary Neolithic groups^4–6^. In the later Chalcolithic period, evidence of distinctive cultural practices and associated population movements highlight the dynamic history of the region, especially in the Southern Levant^7,8^. Further, genomic studies from the Bronze to Iron Ages in the Levant also report admixture and population movements, suggesting some degree of continuity with modern populations^6,9–11^. On a more recent timescale, studies from the medieval period^12^ and modern populations^13,14^ describe genetic structure and the role played by culture and religion in the formation of these structures. Notably, a study on medieval individuals from Lebanon previously identified as Crusaders^12^ demonstrated their ancestry to be either European or local as well as an admixture between Europeans and Near-Easterners. These signals cannot be detected in modern Lebanese groups, suggesting that they were only transient. This provided evidence of genetic signatures of historical religious events such as The Crusades which saw considerable movement from Europe to the Middle East, admixture with local populations and eventually, their dilution over time. Genetic analyses of modern Lebanese populations^13^ suggest that population movements linked to the spread of religions like Islam in the past millennium led to stratification in the Levant. Further south, data from modern Yemenis combined with other Middle Eastern groups found little correlation between genetic structure and geography^14^. It is thus clear that the present distribution of genetic diversity in the Middle East is the result of convoluted processes with culture as an additional level of complexity.

The Late Antiquity period, roughly defined as the time between the third–eighth centuries, was a time of cultural and religious upheaval in the Middle East associated with the emerging Arab Islamic empire. Byzantine Syria-Palestine represents an instance of a region that was conquered by Islamic Arabs in the first half of the seventh century AD (630 s). This area became the political centre of the empire with the founding of the Umayyad caliphate in Damascus in 661^15^. However, the Arabization and Islamisation of the area did not fully take place until the last decade of the seventh century led by ‘Abd al-Malik^16^. As such, the Aramaean, Byzantine and Christian legacy interacted with the new Arab Islamic rule and cultural values for decades. The collapse of the caliphate (750) and transfer of the political centre to Iraq caused political marginalisation and economic decline in Syria-Palestine^17^ (see also Supplementary Note S1 – Early Islamic Southern Syria). Thus within the span of the Umayyad caliphate, this region likely mirrored some of the many political and religious shifts occurring throughout the Middle East^17^.

Ancient DNA analysis is a powerful tool to provide a genomic snapshot of this dynamic period, giving insight into past demographic processes of a currently conflicted and inaccessible territory of the Levant. However, while much of the focus of Near-Eastern Archaeology has been on funerary remains, few Islamic burials have been investigated as such studies might be considered as harming or disturbing the dead. We present an archaeogenomic analysis of two Umayyad Era individuals found at a prehistoric site in modern-day Syria, with no connection to an Islamic cemetery, but buried with indications of Islamic funerary rituals (see Supplementary Note S1 – History of the excavation and subsequent bioarchaeological analysis – for the details of the circumstances of the excavation). We find genomic similarities to a group of modern-day Bedouins and Saudi rather than to most neighbouring Levantine groups. The remains are curated by the General Directorate of Antiquities and Museums (DGAM) of the Arab Republic of Syria and their genomic study is an invaluable resource to understand their ancestry and the history of this region.

## Results and discussion

### Two historic burials on top of a Neolithic site

**Fig. 1:**
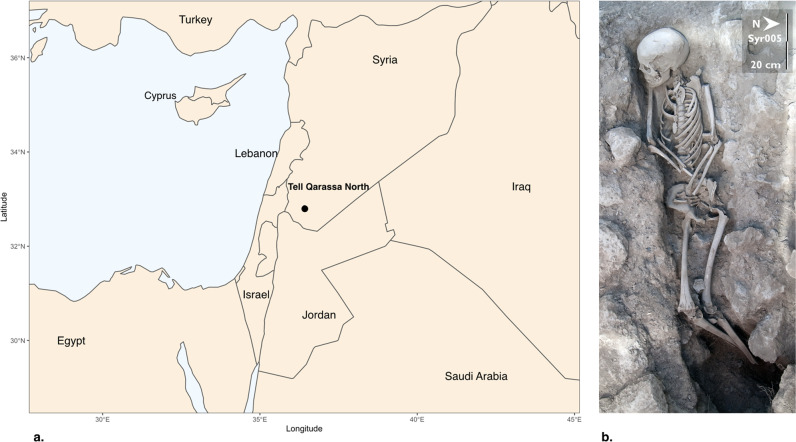
Site location and skeletal remains included in the analysis. **a** Map of the Levant indicating the location of Tell Qarassa in South Syria. **b** Skeletal remains of syr005 during the excavations at Tell Qarassa (Photo by Jonathan Santana).

In this study, we perform genomic analyses of two buried individuals excavated at Tell Qarassa North, a Neolithic site in the Village of Qarassa in Syria (Fig. 1a). While Tell Qarassa North is usually known as a prehistoric site^18,19^ (see also Supplementary Note S1), the two individuals analysed here were found in two narrow graves on surface levels of the site and directly radiocarbon dated to the Umayyad Era (seventh to eighth centuries) (Table 1). No cultural artifacts were associated with the human remains and no evidence of an Umayyad Era cemetery was documented at the site. The two Umayyad Era burials (UEB) were located very close to each other. While in the Neolithic burials the bodies are placed in a flexed position, these bodies were placed in a decubitus position, oriented east-west, with the head at the west, facing south inside pits that were intrusive in the Neolithic levels (Fig. 1b). The distribution of the skeletal elements suggests that both bodies were wrapped before burial^20^. Individual syr005 was a 14–15-year-old male (Fig. 1b) and individual syr013 was a female of about 15–21 years at the time of death (Table 1). Age-at-death estimation was based on the following criteria: pattern of dental eruption, synostosis of epiphyses on the long bone and closure of the sternal ends of the clavicles^21–23^. Together with the radiocarbon dates, the wrapping, the position and orientation of the bodies facing Mecca are concordant with Muslim funerary rituals following Early Islamic burials^24^. However, these individuals were not buried in a traditional Muslim cemetery. This may be explained due to special circumstances of death or cultural identity: nomadic populations, pilgrims, deviant burials or plague victims. The requirement of a Muslim burial to take place within 24 h after death might have made some compromises necessary. It is known that one of the defining features of Muslim burials is that of only one person per grave, which implies that husbands and wives are not buried together, and collective family tombs are forbidden. Nonetheless, occasionally and in extreme circumstances this can be relaxed for victims of plague or warfare (Supplementary Note S1 – Muslim Burials). Also, the close proximity of radiocarbon dates for syr005 (1294 ± 18 Cal BP) and syr013 (1302 ± 15 Cal BP) suggest that both individuals died at a similar time.

**Table 1:** Information about the two sequenced samples.

Individual	Genome coverage	mt coverage	mt haplogroup	Y haplogroup	Biological sex	Age (C14 cal yBP, 2σ)	Autosomal contamination estimate
syr005	0.159799	23.5158	J2a2a1a1	J	XY	666–768 cal AD	0.01475
syr013	6.15177	522.349	R0a2	-	XX	665–766 cal AD	0.0488

### Genome sequencing and exploratory analysis

To investigate the genetic identity of these two individuals, their association to past, contemporaneous and present-day Middle Eastern populations as well as to shed light on past genetic variation of Syria, a conflicted region that remains currently poorly studied, we shotgun-sequenced two petrous bones (syr005 and syr013) at a depth coverage of 0.16× and 6.15×, respectively (Table 1). Sequence data from both individuals showed characteristic patterns of post-mortem damage and fragmentation expected from endogenous ancient DNA (aDNA) molecules^25^ (Fig. S1). We used four different methods to estimate contamination at the mitochondrial^25,26^, autosomal^27^ and X-chromosome^28^ levels and all four methods confirmed low levels of contamination (<5%, Table S2). Two biological sex inference methods^29,30^ identified syr005 to be a male and syr013 a female. Individuals syr005 and syr013 were determined to carry mitochondrial haplogroups J2a2a1a1 and R0a2, respectively. Both haplogroups are common in the Arabian Peninsula, Near East and parts of Africa^31,32^ in concordance with the broad geographical location of the samples. In addition, the Y chromosome of syr005 was determined as haplogroup J, which is the most common haplogroup across the Middle East^33^ (Table S3).

**Fig. 2:**
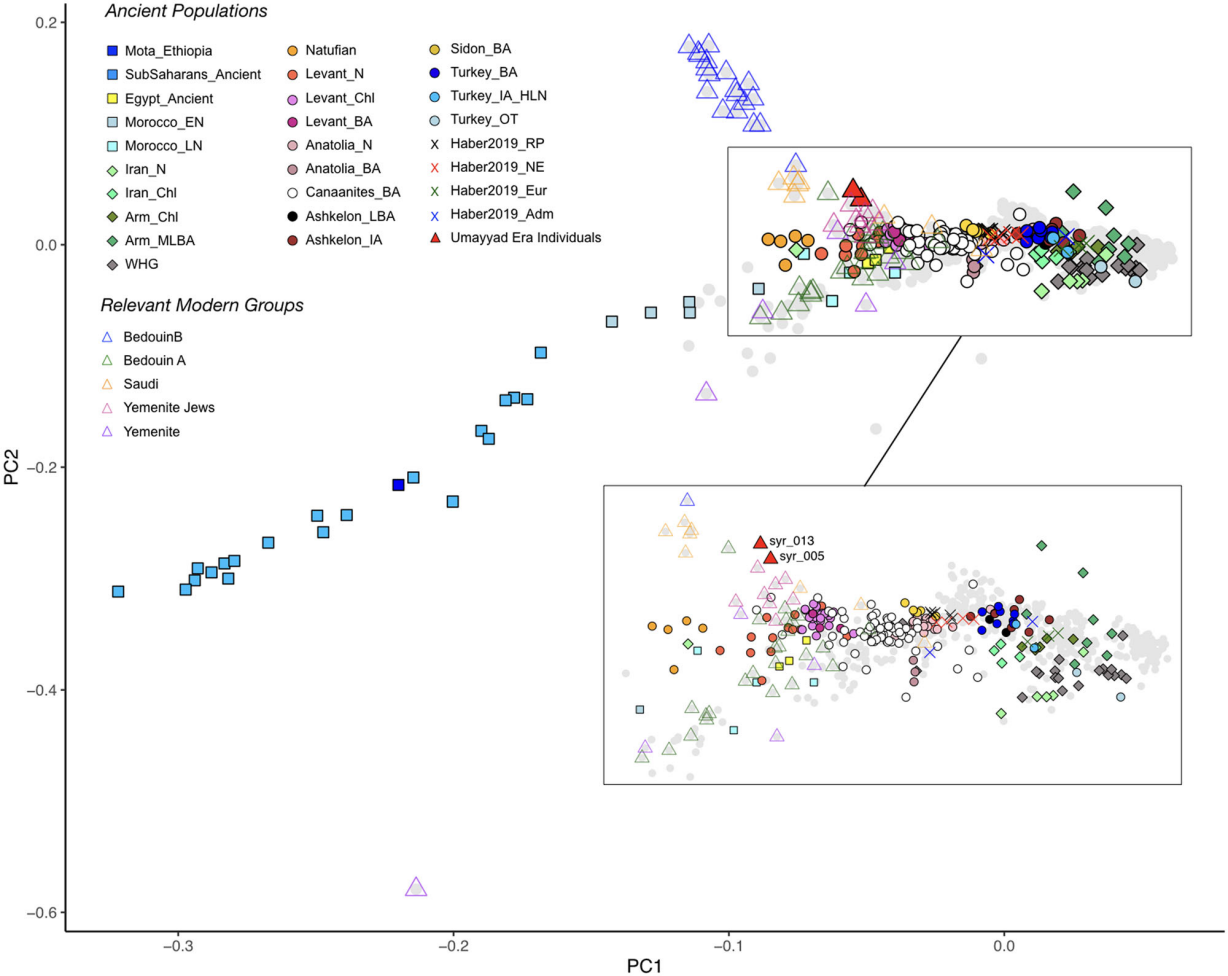
Principal component analysis of modern and ancient Middle Eastern populations. PCA with ancient Levantine populations projected on modern Middle Eastern genetic variation (grey), with some relevant groups indicated as open triangles.

To further explore general patterns of genetic affinity to modern populations, we performed a principal component analysis (PCA), projecting the two newly sequenced Umayyad Era individuals along with 262 published ancient individuals from the Near East, Western Europe, North and sub-Saharan Africa on a broad set of modern Middle Eastern, Arabian Peninsula, European and North African groups. The two UEB individuals fell between modern genetic variation in the Middle East and Arabian Peninsula and are shifted towards the latter (Fig. S2). Further, to obtain a better understanding of the regional variation, we conducted a second PCA, limited to 37 modern groups from the Middle East, Arabian Peninsula and Caucasus (Fig. 2). While the UEB individuals did not cluster with any published ancient Levantine individuals, the closest ancient groups were Bronze Age Canaanites^8^ and groups from Neolithic, Chalcolithic and Bronze Age Levant^1,7^. Among modern human populations, the UEB individuals are positioned between groups known to originate from/inhabiting the Arabian Peninsula, i.e. Saudi, Yemenite Jews and Bedouin A and B (Fig. 2). Hence, overall, individuals syr005 and syr013 fall between the two Bedouin groups, and show a clear genetic differentiation from other modern-day Levantines.

**Fig. 3:**
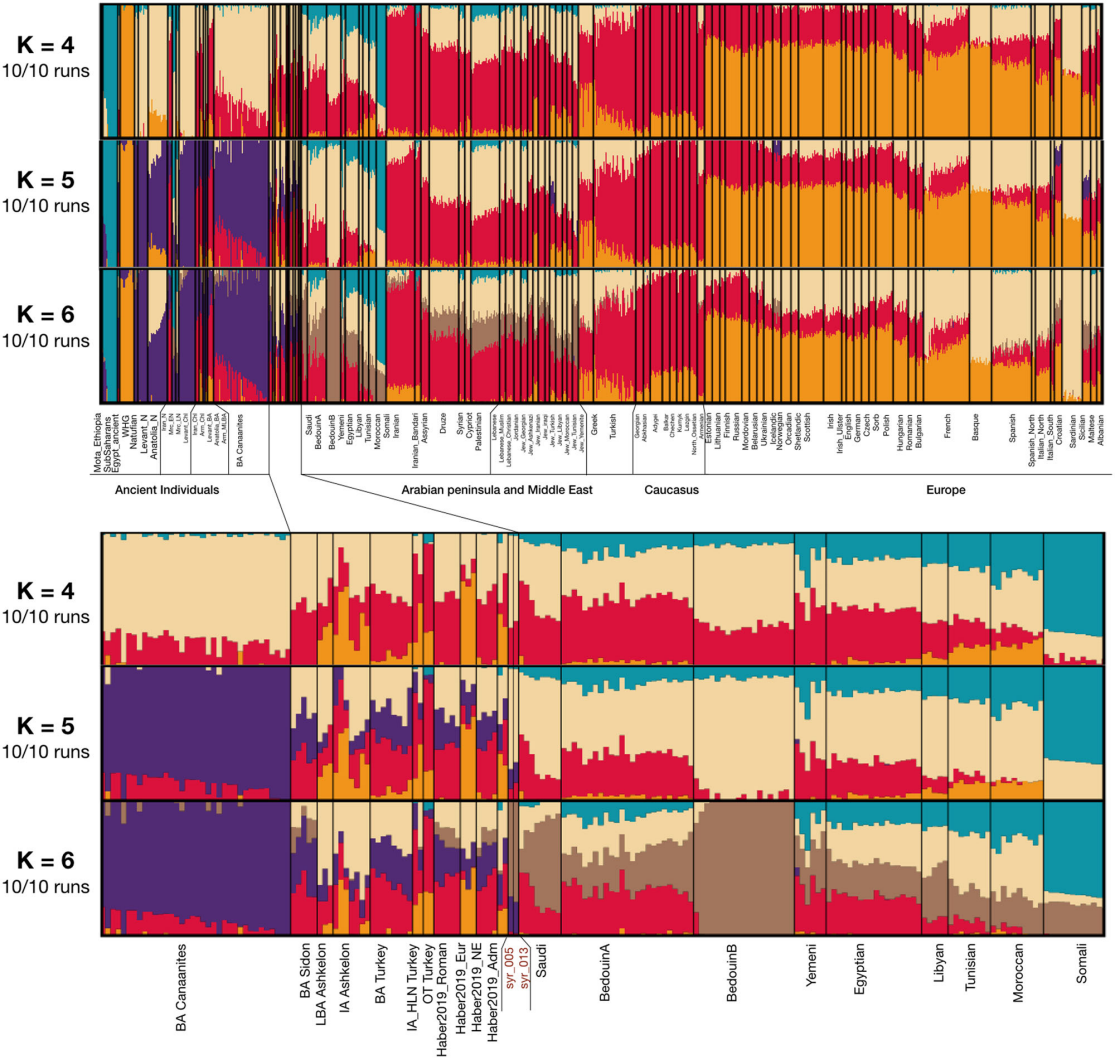
Model-based clustering of ancient and modern populations. *ADMIXTURE* run with ancient individuals and present-day modern groups. The zoomed in figure shows ancestry proportions of the UEB individuals along with some ancient and modern populations at K = 4, 5, 6.

To gain insight into the genetic composition of ancient and modern populations, we ran an unsupervised *ADMIXTURE*^34^ analysis (Fig. 3) with a set of 1321 individuals from Europe and the Middle East (73 modern and 28 ancient populations in total). For K = 2, 4, 5 and 6, all iterations with different random seeds converged to consistent results (Fig. S3). Therefore, we consider K = 6 as a compromise between the resolution and robustness of the results. At K = 4, a new component appeared mostly in prehistoric Levantine groups, i.e. Natufians, Neolithic Levant, Neolithic Anatolia, Chalcolithic Levant and in the later Bronze Age Canaanites^8^ as well as in the UEB individuals. This was also seen in moderate proportions across modern groups from the Arabian Peninsula/Middle East, and in lower proportions in some European groups. At K = 5, this component split up in two, one appeared exclusive to ancient Levantine groups (as in K = 4) but only in low amounts in the UEB individuals and was absent in any modern population from the Middle East/Arabian Peninsula which showed high values of the second component maximised in Bedouin B. At K = 6, another component emerged in Bedouin B and was present in high to moderate proportions in groups like Saudi/Bedouin A/Yemenite Jews and Middle Eastern populations, respectively. The UEB individuals also harbour high proportions of this component along with small amounts of the Neolithic Levant component seen at K = 5.

**Fig. 4:**
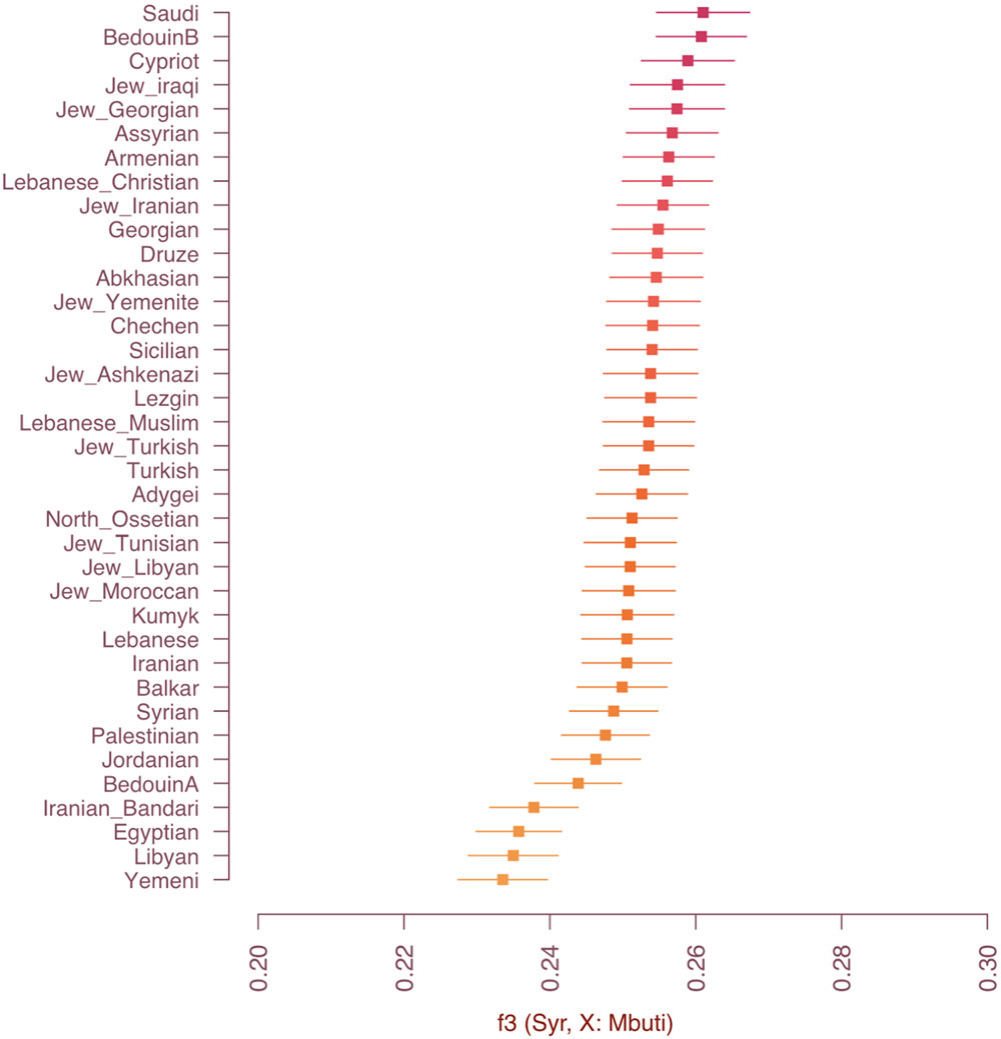
Affinity to modern populations. Outgroup f_3_ statistics show shared genetic drift between the UEB individuals and modern-day populations. Standard errors (SE) were estimated using a weighted block-jackknife procedure. Error bars represent two SEs.

**Fig. 5:**
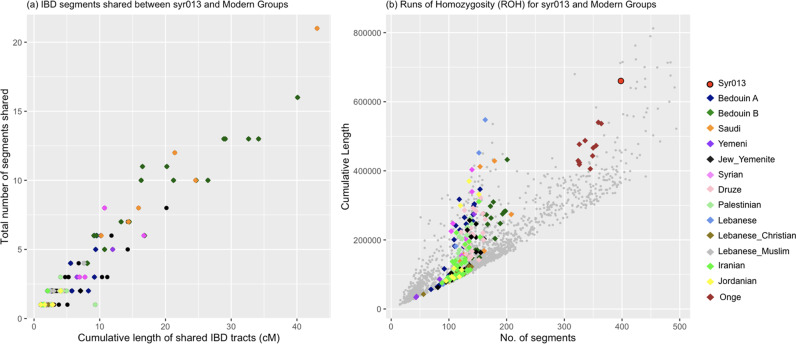
Analysis of diploid genotype calls. **a** Shared IBD tracts between syr013 and modern populations from the Human Origins 2.0 dataset. The X-axis indicates the cumulative length (in centiMorgans) of shared tracts between a pair consisting of syr013 and a modern-day individual, while the Y-axis shows the total number of shared segments for that pair. Relevant populations are indicated as coloured diamonds. **b** Plot showing Runs of Homozygosity (ROH) for syr013 with relevant modern populations. The X-axis indicates the number of segments with ROH and the Y-axis is their cumulative length.

We conducted outgroup *f*_*3*_ statistics^35^ to increase resolution on population affinities between the two UEB samples and modern Bedouins, Saudi and Yemenite Jews, as indicated by PCA and *ADMIXTURE* analysis. We observed high shared genetic drift with Bedouin B and Saudi (Fig. 4), but given the similar values of the statistic (*f*_*3*_*(X, UEB individuals; Mbuti)*, Table S4), establishing which modern group has the highest affinity required further analyses. To fully harness the medium coverage data for syr013, we called diploid genotypes using a genotyper designed for ancient DNA (*snpAD*^36^). Compared to the pseudohaploid data available for the other ancient individuals, these diploid genotypes allowed for a more fine scale analysis of the relationship between syr013 and modern populations. We used Beagle 4.1^37^ to analyze sharing of tracts of identity by descent (IBD) between syr013 and present-day populations. Consistent with other results, the highest number and total length of IBD tracts was shared with multiple Bedouin B individuals and a single Saudi individual in the Human Origins 2.0 dataset (Fig. 5a). This confirms the connection between the UEB individuals and nomadic Levantines as well as to the Arabian peninsula.

We then tested different scenarios to investigate the most likely population affinities between the sequenced UEB samples and modern regional populations, performing D statistics of the form *D(UEB, A; X, Mbuti)* testing whether ‘X’ is an outgroup with respect to the group constituted by UEB individuals and ‘A’ or if there is an excess of allele sharing between ‘A’ and ‘X’ or the UEB individuals and ‘X’. When we tested Bedouin B and Saudi as candidates for groups ‘A’ and ‘X’, we found that no population configuration using this topology was consistent with the data (Tables S5–S8). Bedouins B were rejected as a sister group to the UEB individuals to the exclusion of Saudi due to an excess of allele sharing between Saudi and the UEB (*Z = 6.4*) while Saudi were rejected as a sister group due to an excess of allele sharing between the UEB and Bedouin B (*Z = 13.8*). Testing the UEB individuals as an outgroup to Saudi and Bedouin B revealed this to be a scenario consistent with the data (*Z = 0.19*) confirming that the two UEB individuals form their own group and are not directly matched by any of the modern populations in our reference data.

### Modelling the genomic ancestry of the UEB individuals

From a genetic perspective, Levantine populations today fall into a continuum of genetic ancestry, consisting of varying proportions derived from different prehistoric populations. Recent studies suggest differences in the genomic history of the Levant and the Arabian Peninsula, with higher ancient Levantine, Iranian and Eurasian Hunter-Gatherer ancestry proportions in the Levant and excess African ancestry in the latter^14,38^. Within the Levant itself, groups such as Syrians, Palestinians and Jordanians have also been suggested to have higher African ancestry relative to nearby populations^13,14^ (see also Table 2). To obtain a deeper understanding of these different ancestries, we used *qpAdm* to explore different scenarios for the UEB individuals and relevant modern and historical Levantine populations.

**Table 2:** Modelling ancient and modern Middle Eastern populations.

*2-source models*					
	*p* value	Levant_N	Iran_N	WHG	
UEB Individuals	0.289238	0.610 ± 0.060	0.390 ± 0.060	NA	
UEB Individuals_dmg	0.437987	0.666 ± 0.069	0.334 ± 0.069	NA	
Turkey_IA_HLN	0.152261	0.515 ± 0.079	0.485 ± 0.079	NA	
Mrc_LN	0.0880385	0.721 ± 0.096	0.279 ± 0.096	NA	
Levant_BA	0.116881	0.784 ± 0.031	0.216 ± 0.031	NA	
Haber2019_NE	0.0687897	0.723 ± 0.049	0.277 ± 0.049	NA	
Ash_LBA	0.0636178	0.640 ± 0.131	0.360 ± 0.131	NA	
Ash_LBA	0.085684	0.787 ± 0.069	NA	0.213 ± 0.069	
*3-source models*					
	***p*** **value**	**Levant_N**	**Iran_N**	**WHG**	**Mota_Ethiopia**
UEB Individuals	0.396026	0.598 ± 0.058	0.348 ± 0.065	0.055 ± 0.038	NA
UEB Individuals_dmg	0.651473	0.655 ± 0.065	0.281 ± 0.074	0.064 ± 0.043	NA
Bedouin B	0.112744	0.552 ± 0.026	0.394 ± 0.030	NA	0.054 ± 0.007
Saudi	0.224519	0.534 ± 0.028	0.435 ± 0.033	NA	0.031 ± 0.008
Jew_Yemenite	0.158269	0.557 ± 0.027	0.402 ± 0.031	NA	0.041 ± 0.008
Lebanese_Christian	0.309808	0.599 ± 0.020	0.321 ± 0.024	0.080 ± 0.013	NA
Lebanese_Muslim	0.249849	0.488 ± 0.021	0.459 ± 0.026	0.052 ± 0.014	NA
Druze	0.394225	0.543 ± 0.019	0.383 ± 0.023	0.073 ± 0.013	NA
Cypriot	0.105134	0.655 ± 0.021	0.243 ± 0.025	0.103 ± 0.013	NA
Haber2019_NE	0.196697	0.704 ± 0.048	0.233 ± 0.053	0.063 ± 0.031	NA
Ash_IA	0.213846	0.531 ± 0.068	0.328 ± 0.083	0.141 ± 0.043	NA
Ash_LBA	0.164834	0.613 ± 0.119	0.241 ± 0.142	0.145 ± 0.075	NA
Levant_BA	0.866914	0.784 ± 0.030	0.169 ± 0.035	0.046 ± 0.018	NA
Turkey_IA_HLN	0.889632	0.463 ± 0.072	0.407 ± 0.076	0.130 ± 0.051	NA
Mrc_EN	0.0906708	0.327 ± 0.079	0.335 ± 0.089	NA	0.338 ± 0.023
Mrc_LN	0.604079	0.804 ± 0.100	0.120 ± 0.117	NA	0.076 ± 0.031
*4-source models*					
	***p*** **value**	**Levant_N**	**Iran_N**	**WHG**	**Mota_Ethiopia**
UEB Individuals (infeasible)	0.87482	0.564 ± 0.061	0.426 ± 0.080	0.043 ± 0.040	−0.032 ± 0.020
UEB Individuals_dmg (infeasible)	0.671526	0.639 ± 0.066	0.326 ± 0.090	0.055 ± 0.045	−0.021 ± 0.020
Bedouin B	0.182317	0.568 ± 0.027	0.343 ± 0.043	0.027 ± 0.016	0.062 ± 0.009
Saudi	0.561657	0.551 ± 0.028	0.376 ± 0.044	0.032 ± 0.017	0.041 ± 0.009
Jew_Yemenite	0.672415	0.579 ± 0.027	0.332 ± 0.044	0.036 ± 0.017	0.053 ± 0.009
Yemeni	0.895286	0.441 ± 0.027	0.334 ± 0.042	0.048 ± 0.016	0.177 ± 0.009
Bedouin A	0.430541	0.517 ± 0.022	0.316 ± 0.036	0.058 ± 0.015	0.109 ± 0.008
Syrian	0.589049	0.485 ± 0.026	0.380 ± 0.042	0.075 ± 0.016	0.060 ± 0.009
Lebanese	0.770664	0.527 ± 0.025	0.350 ± 0.040	0.080 ± 0.016	0.044 ± 0.009
Lebanese_Christian (infeasible)	0.172228	0.595 ± 0.025	0.329 ± 0.040	0.078 ± 0.016	−0.002 ± 0.008
Lebanese_Muslim	0.606834	0.515 ± 0.025	0.402 ± 0.040	0.069 ± 0.016	0.015 ± 0.008
Jordanian	0.858381	0.514 ± 0.024	0.334 ± 0.038	0.081 ± 0.015	0.072 ± 0.008
Druze	0.254556	0.551 ± 0.025	0.367 ± 0.040	0.078 ± 0.015	0.004 ± 0.008
Cypriot (infeasible)	0.31197	0.625 ± 0.026	0.308 ± 0.043	0.084 ± 0.017	−0.017 ± 0.009
Palestinian	0.689973	0.535 ± 0.023	0.335 ± 0.037	0.063 ± 0.014	0.066 ± 0.008
Haber2019_NE (infeasible)	0.648016	0.668 ± 0.050	0.309 ± 0.065	0.053 ± 0.032	−0.031 ± 0.016
Haber2019_RP (infeasible)	0.00555823	0.673 ± 0.052	0.298 ± 0.071	0.034 ± 0.032	−0.004 ± 0.015
Haber2019_Eur (infeasible)	0.0305447	0.390 ± 0.057	0.308 ± 0.072	0.365 ± 0.038	−0.063 ± 0.017
Haber2019_Adm (infeasible)	0.0282109	0.605 ± 0.053	0.230 ± 0.072	0.188 ± 0.035	−0.022 ± 0.017
AgTam_BACanaanites (infeasible)	0.53806	0.712 ± 0.026	0.273 ± 0.042	0.040 ± 0.016	−0.025 ± 0.008
Anatolia_BA (infeasible)	0.139901	0.801 ± 0.045	0.244 ± 0.073	0.014 ± 0.026	−0.059 ± 0.014
Arm_MLBA (infeasible)	0.0577635	0.331 ± 0.078	0.595 ± 0.110	0.120 ± 0.047	−0.046 ± 0.022
Arm_Chl (infeasible)	0.940145	0.488 ± 0.032	0.408 ± 0.050	0.152 ± 0.021	−0.048 ± 0.010
Ash_IA	0.112139	0.555 ± 0.109	0.286 ± 0.172	0.149 ± 0.052	0.010 ± 0.035
Ash_LBA	0.0944184	0.672 ± 0.199	0.105 ± 0.375	0.190 ± 0.136	0.033 ± 0.074
Iran_Chl (infeasible)	0.598333	0.429 ± 0.037	0.623 ± 0.059	−0.014 ± 0.021	−0.038 ± 0.011
Levant_Chl (infeasible)	0.239445	0.919 ± 0.032	0.117 ± 0.052	−0.004 ± 0.020	−0.032 ± 0.010
Levant_BA (infeasible)	0.738578	0.775 ± 0.040	0.189 ± 0.066	0.041 ± 0.024	−0.004 ± 0.013
Mrc_EN	0.202773	0.306 ± 0.075	0.252 ± 0.097	0.089 ± 0.049	0.352 ± 0.024
Mrc_LN (infeasible)	0.457194	0.812 ± 0.102	0.153 ± 0.133	−0.036 ± 0.070	0.071 ± 0.033
Sidon_BA (infeasible)	0.102462	0.663 ± 0.048	0.355 ± 0.066	0.014 ± 0.033	−0.032 ± 0.015
Turkey_BA (infeasible)	0.0119186	0.661 ± 0.050	0.375 ± 0.064	0.028 ± 0.030	−0.064 ± 0.014
Turkey_IA_HLN (infeasible)	0.86774	0.451 ± 0.076	0.437 ± 0.093	0.125 ± 0.052	−0.013 ± 0.023
Turkey_OT (infeasible)	0.0194304	0.107 ± 0.072	0.596 ± 0.098	0.254 ± 0.051	0.042 ± 0.022

This analysis used Neolithic Levant (Levant_N), Neolithic Iran (Iran_N), European Western Hunter-Gatherers (WHG) and Mota from Ethiopia as source populations. Nested two-source and three-source feasible models found in the four-source scenario are shown first, followed by the results of ancestry proportions obtained from all four sources. For three-sources, NA implies that that source was not used in the model. Values are shown along with standard errors, with infeasible models pointed out along with the target population. Outgroups used: Mbuti, Sweden Hunter-Gatherers (SHG), Kostenki14, Ust-Ishim, Central Hunter-Gatherers (CHG) and Neolithic Anatolia. UEB_dmg indicates models run with data from the UEB individuals restricted to damaged sites.

**Fig. 6:**
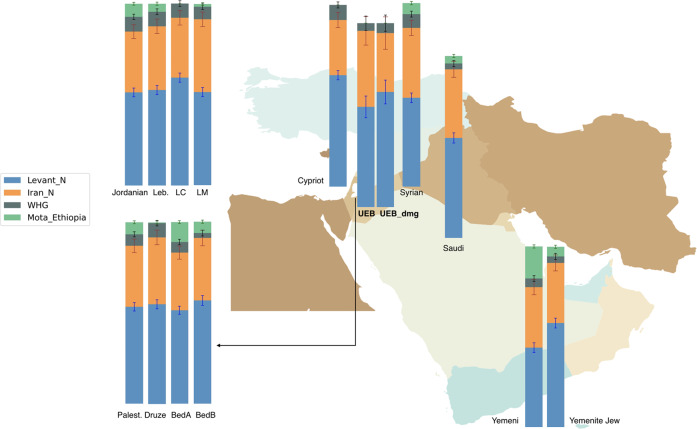
Ancestry modelling of Levantine populations. Ancestry of a smaller subset of Middle Eastern populations modelled from Neolithic Levant, Neolithic Iran, European Hunter-Gatherers (WHG) and a 4500-year-old individual Mota from Ethiopia using *qpAdm*. Ancient populations include the UEB individuals^12^ (Palest. Palestinians, Leb. Lebanese, LM Lebanese Muslim, LC Lebanese Christians, BedA,B Bedouin A, B). Error bars represent one standard error.

We used Neolithic Levant, Neolithic Iran, Western European Hunter-Gatherers and ancient East Africans approximated by Mota (a 4500-year-old individual from Ethiopia^39^) as potential sources of ancestry. We found that most ancient groups do not require all four sources, but that few of them can always be modelled as a mixture of Levant_N, Iran_N and either WHG or Mota, (except Late Bronze Age individuals from Ashkelon^10^ which can be modelled without Iran_N). The UEB individuals can be modelled either as a two-way mixture of Levant_N (61 ± 6%) and Iran_N (39 ± 6%) (Table 2), or also with a minor contribution of ancestry from WHG (6 ± 4%) but no models work with Mota as a source (Table 2). Interestingly, Bedouin B, Saudi and Yemenite Jews could be modelled from all four sources, i.e. Levant_N (56.8 ± 2.7%, 55.1 ± 2.8% and 57.9 ± 2.7%), Iran_N (34.3 ± 4.3%, 37.6 ± 4.4% and 33.2 ± 4.4%), WHG (2.7 ± 1.6%, 3.2 ± 1.7% and 3.6 ± 1.7%) and Mota (6.2 ± 0.9%, 4.1 ± 0.9% and 5.3 ± 0.9%) as well as from only Levant_N, Iran_N and Mota (see Table 2). In addition, most modern Levantine populations except Lebanese Christians and Cypriots can be shown as a mixture of all four sources (Fig. 6).

Thus, despite indications from other analyses that Bedouin B and Saudi seem to be the closest modern populations to the UEB individuals, we obtained slightly different results from qpAdm analyses since these modern groups required East African ancestry for a fitting model. This lack of African-related ancestry in the UEB individuals in contrast to their closest modern populations might be the cause of the D statistics results (which placed the UEB individuals as an outgroup to Saudi and Bedouin B) and could be explained by different scenarios. For instance, the Neolithic Levantine ancestral background could have been diluted by the introduction of African-related ancestry later, i.e. after the UEB individuals. This might also account for the observation that none of the other historical Levantine individuals from earlier time periods seem to produce a fitting model with Mota as an ancestral source. In fact, the only ancient individuals that could be successfully modelled with African-related ancestry are Neolithic Moroccans^40^. Consistent with this explanation, when we also tried to model the UEB individuals as a two-way mixture of already published Levantine individuals more proximate in time and Mota, we obtained high standard errors for Mota-related ancestry in all models and found only a few successful single-source models from published Levantine individuals (Table S9). Further, when we tried to model modern Levantine populations as a mixture of UEB individuals and Mota (Table S10), we found that all populations required a high amount of ancestry from UEB individuals (~80–99%) and variable contributions from Mota (0.1–20%).

It is also possible that the UEB individuals are representative of groups that migrated from the Arabian Peninsula to the Levant during the early years of Islam and experienced strong cultural barriers that over time prevented mixing with neighbouring populations, resulting in the highly drifted population observed in our data. This would be concordant with the fact that the ancestors of the genetically similar genotyped Negev Bedouins migrated from the Arabian Peninsula to the Negev and Sinai regions around 700 CE, i.e. shortly after the spread of Islam^41^. However, population-level genetic and archaeological data are required to make conclusive inferences about large-scale migrations and religious/cultural stratification especially since it is possible that the Arabian peninsula-related ancestry was present in the Levant long before the UEB individuals lived. It is worth noting that the historical UEB individuals are the population with the lowest amount of genomic data, restricting a precise estimation or even a full rejection of the presence of European or African ancestry in these individuals, thus affecting ancestry estimates. However, even if we cannot fully reject the presence of any African ancestry, all analyses consistently suggest a lower level of African ancestry in the UEB individuals. While the reduced resolution for the historical UEB individuals prevents us from making more reliable inferences at this stage, the genetic differences observed here could potentially suggest a slightly different trajectory and contacts with other groups in the long term compared to Saudi and Bedouins as well as the effect of recent history on population structure. Similar results were also obtained from models repeated with data from UEB individuals restricted to damaged sites (Tables S9, S10).

### Low genetic diversity in the UEB individuals

In order to gain insight into the genetic diversity of the UEB individuals, we estimated Conditional Nucleotide diversity (CND)^42^ to be 0.205 ± 0.003. When compared to all modern Middle East populations in our reference panel^1^, syr005 and syr013 showed the lowest levels of genetic diversity. For comparison purposes, we included Onge (native hunter-gatherers from the Andaman Islands known to have extremely low genetic diversity due to their small population size and prolonged isolation^43^) and results showed a lower value of CND than syr005 and syr013 (Fig. S4 and Table S11). However, it should be noted that comparisons between ancient and modern populations, sequence and SNP chip data might be subject to some biases^42,44,45^. Despite this low genetic difference between the two individuals, we do not see evidence of them being close relatives as the between individuals distance is almost exactly twice the within an individual distance (using a pseudo-twin representation of syr005 and syr013, see Methods) as expected for an unrelated pair of individuals. As low genetic diversity could be the result of small population size and/or inbreeding between individuals, we analysed runs of homozygosity (ROH) to determine the extent of inbreeding. In the syr013 individual, we observe both high numbers and cumulative lengths of ROH segments in syr013 compared to other Middle Eastern populations (Fig. 5b). Consistent with CND results, this suggests that syr013 was part of a small and/or inbred population. To ensure that the results were not affected considerably by missing sites, we also ran the analysis with the Simons Genome Diversity Panel (SGDP)^46^ restricting the analysis to the sites covered for syr013 and found a similar pattern (Fig. S5). These findings also might suggest similar social structures as those seen in nomadic groups in the Levant, where affiliation of individuals to tribal or clan-structured groups is associated with consanguinity, strong barriers to extra-tribal marriage and results in low genetic diversity, small effective population size and a high incidence of recessive disorders^47,48^.

### Insights into the phenotype of the UEB individuals

To investigate potential lactase persistence in Umayyad Era Syria we tested five SNPs known to be associated with the trait (Table S12). All five SNPs analysed here are located upstream of the gene *LCT* (encoding lactase) in introns of the gene *MCM6*, which serves as an enhancer for *LCT* transcription:^49^ −13910C/T, −13915T/G, −14010G/C, −13907C/G, and the rare variant −14107G/A. Lactase persistence is known to be an autosomal dominant trait, thus the presence of a single derived allele is sufficient for milk digestion. Interestingly, nine reads in sample syr013 were found mapping to the SNP −13915T/G, out of which five were the derived allele and four were ancestral which indicates that syr013 was heterozygous and lactose tolerant. Despite the long history of camel herding in this region beginning with its domestication more than 6000 years ago^50^, syr013 being heterozygous marks the earliest observation of this variant in genomic data. Lactase persistence could not be tested for sample syr005 as no reads covered the sites of interest. Nevertheless, this finding again draws a connection to the Arabian Peninsula as this variant is common in modern Arabian (for e.g. has a frequency of 72–88% in Saudi^50^) populations as well as in pastoralists that have traditionally relied on the Arabian camel (*Dromedary camelous*) for milk consumption and show high levels of lactase persistence phenotypes (>75% of individuals in Bedouins)^49,50^. We also tested other autosomal dominant or recessive conditions reported to occur frequently in Arabs^47^ (including familial hypercholesterolaemia, glucose-6-phosphate dehydrogenase deficiency, sickle-cell anemia, Bardet-Biedl syndrome, etc.) but no pathogenic alleles were found in syr013 (Table S13).

### Diet of the UEB individuals

We attempted to infer dietary consumption patterns in these individuals using stable isotope analysis, using collagen samples extracted from the petrous bone (Table S14). The bulk stable isotope data for these individuals are typical of a C_3_ terrestrial diet (δ^13^C −18.5‰ and −19.2‰; δ^15^N + 11.5‰ and +13.1‰) with high animal protein intake. Consumption of food from an arid location or where manuring was practiced are also possible explanations for the high nitrogen values. Without comparative faunal or human data from the period and location, it is difficult to ascertain the extent of these effects. We contextualised our data with human stable isotope regional datasets from historical/proto-historical periods (e.g. from Lebanon, Jerusalem, Northern Jordan, Syria [albeit Northern], including Early and Late Roman, Parthian, Byzantine, Medieval and some modern data^51–55^ (see Table S14 and Fig. S6). In addition, we have compared this data with those obtained from Bedouin tombs from Jordan dating between the 13th and 19th centuries^56^. The regional datasets demonstrate similar δ^13^C values to the two UEB individuals studied here, but generally lower δ^15^N (6.8–9.5‰, (excluding St Stephens, Jerusalem)), with the exception of a Medieval individual with European ancestry (δ^15^N = 11.3‰) excavated from Lebanon^12^. It is possible that this individual hailed from Europe and had a different diet from those in the Middle East (before being buried there). The monastic community of St Stephens tends to have higher nitrogen values than the other datasets δ^15^N (7.3–12.6‰ and 6 individuals of the 68 as high or higher than 11.5‰). The high nitrogen values for this site have been attributed to animal protein consumption^51^. The Bedouin dataset has comparable (and higher) nitrogen isotope ratios to our two individuals (δ^15^N 10–17.3‰), however, less negative δ^13^C values. The high nitrogen values of the Bedouin are attributed to 'considerable amounts of animal protein' including blood, milk (products) and meat, and the high δ^13^C ratios are attributed to the habitation of and resource exploitation of predominantly C_4_ or mixed C_4_-C_3_ ecosystems^56^. Compound specific amino acid carbon isotope values were obtained^57,58^ to investigate whether the high nitrogen values were influenced by the intake of aquatic resources. The δ^13^C_GLY_-δ^13^C_PHE_ values for the individuals are between 10 and 13‰^59^, δ^13^C_VAL_-δ^13^C_PHE_ values around 0 to 1‰^59,60^ and the δ^13^C_LEU_-δ^13^C_PHE_ values are negative (Table S15); these values are consistent with a terrestrial diet without significant input from aquatic resources^58,61^. We conclude that the Tell Qarassa individual’s isotope values can best be explained as diets with high levels of animal product consumption (i.e. pastoral food acquisition – like the Bedouin dataset), but obtained from an almost exclusively C3 ecosystem (like the other regional datasets).

The majority of stable isotope data discussed herein were measured from collagen samples extracted from cortical long bone and ribs, with the exception of four samples taken from molars^53^ and the data from refs. ^11,12,52^, who, like us, used petrous bone. Jørkov et al.^62^ have argued that the isotope signal from petrous bone reflects some childhood diet, reporting slightly lower δ^13^C values (<0.3‰) and slightly higher δ^15^N values (<0.8‰) in petrous bone compared to those of rib and femur samples (at a population level). Even if this offset applies to our dataset, these differences would not affect the dietary interpretation of the two individuals from Tell Qarassa.

This dietary interpretation is intriguingly consistent with the Bedouin ancestry components of the individuals and the presence of the lactase persistence variant in syr013. Dietary stable isotope studies of ancient Islamic individuals have been widely studied in Iberia (and the Balearic Islands), but less extensively elsewhere. Lopez-Costas and Alexander^63^ conclude that there is no strong evidence that Islamic diet has a distinct isotopic signal that distinguishes it from contemporary Christian diets (e.g. ref. ^64^), despite some well-known cultural differences (e.g. the prohibition of pork in Islam, fasting and fish-eating in Christianity). They highlight that further research is required to investigate such distinctions^63^ and certainly further work is required in the Middle East to expand our knowledge of dietary stable isotopes in the Umayyad Era.

### Metagenomic screening for pathogens

A possible explanation for both individuals not being buried in a traditional Muslim cemetery could be that they represent plague victims. Several archaeogenomic studies have successfully identified different pathogen sequences in DNA extracted from archaeological remains^65^. We screened our sequences for DNA from different known pathogens and find potential traces of different bacterial species that can cause infections in syr005 (Supplementary Note S2 and Figs. S7–S10). The limited number of sequences does not allow us to unambiguously authenticate these findings and additional research would be required to verify whether a complex infection played a role in the death of these individuals.

## Conclusions

The continuous development and improvement in ancient DNA methodologies and molecular techniques is constantly pushing the temporal and geographic limits of aDNA recovery, exploring older time periods^66–68^ and hostile environments (e.g. hot and humid) for DNA preservation. The Middle East and the Arabian Peninsula are pivotal regions in the timeline of human history and an increasing number of aDNA studies have attempted to understand their genetic history. Although there have been successful studies^1,3–14^, given the poor conditions for DNA preservation, this process is proving to be slower than in more environmentally favourable regions of the world. Nonetheless, given its historical importance, each newly recovered DNA sequence adds an important piece to the genomic and cultural puzzle of a war-stricken region currently under difficult access.

We have been able to infer that the two Umayyad Era individuals buried on top of a prehistoric site represent individuals that were genetically close (while not identical) to a subgroup of modern-day Bedouins ('Bedouin B') from the Negev desert in Israel as well as Saudi from the Arabian peninsula in the Human Origins 2.0 dataset. Several sources document the existence of historical nomadic groups that either occupied the Tell Qarassa region and/or migrated from the Arabian Peninsula to Syria during the Umayyad Era^69,70^. However, the absence of contemporary genetic data from this period limits resolution on finer substructure among such groups. Additionally, while genomic methods are a powerful tool to analyse an individual’s ancestry and infer past demography and population dynamics, genetic datasets often use regional, cultural and/or archaeological affiliations for genotyped individuals. Thus, while it seems that the UEB individuals are genetically similar to some nomadic groups, determining their exact cultural affiliation is a question that cannot be answered through genomic analysis.

The archaeological context proves to be slightly more informative regarding their burial characteristics, i.e. orientation towards Mecca, separate graves and wrapping practice which appears to be indicative of these individuals possibly being early adherents of Islam in a Christian-majority region^71^. The Tell Qarassa graves do not represent a traditional Muslim context, i.e. a traditional Muslim cemetery and – from the available evidence – do not seem to have been located near a permanent settlement of the period. This suggests that it is possible that these two individuals were transient Muslims in the region. The absence of trauma to the bones and the young age of the deceased suggests that they could have died from disease, possibly the Justinian plague which ravaged the Middle East from 541 AD to 749 AD recurring in cycles^72^. Specifically, the dates of the burials may be linked to the outbreak of 79 AH (698 AD) which was reported in Syria by as-Suyuṭī^73^. However, we did not find conclusive evidence of pathogens and the exact cause of their death remains difficult to pinpoint.

The genomic ancestry of the two individuals buried at Tell Qarassa in the late seventh or early eighth century offers a glimpse of early Islamic society in Syria. This study provides further insight into the possible re-use of prehistoric burial sites by Muslim groups. On a general level, this burial provides additional indications for the early adoption of specific Islamic burial rites which were followed even in remote locations. At present, there are no examples of genetic studies from the region which relate to this period, the only genetic data related to early Islamic burials is the study of two individuals from the south of France^74^. Our results suggest the early presence of Muslim Arabs in the Syrian countryside. Extensive additional sampling from different groups in this region is crucial to understand the extent of their genetic structure today and to potentially identify relatively genetically isolated populations, which could have implications for population genetic and clinical studies. The Middle East is a region with a complex history and a diverse ethnic and genetic composition, yet our current understanding of the genetic structure in the past and present appears to have only scratched the surface.

## Methods

### Archaeological context

The archaeological site of Tell Qarassa, located in modern-day Syria (Fig. 1a), is prominently known for its evidence of human occupation since the Epipaleolithic period to the Iron Age^75–77^. Located on the shores of an ancient dried-up lake, it is described to consist of multiple sites or so-called ‘Tells’, one of which (Northern Tell) contains remains from Pre-Pottery Neolithic B to Late Chalcolithic settlements, while the other (Southern Tell) holds evidence of Early Bronze to Iron Age remains. Evidence of a Natufian settlement has also been found in close proximity^78^. The village of Qarassa is a Druze community today. Pre-Pottery Neolithic mortuary practices have been described from this site, shedding light on such practices in this period^18,19^. The individual syr005 was found laid on his back in a decubitus supine position, although the lower limbs were slightly flexed and placed on their right side. The burial was oriented east-west, with the head at the west, facing south. Further, individual syr013 was placed on her right side in a lateral decubitus position oriented east-west, with the head at the west, facing south. The distribution of the skeletal elements suggests that both bodies were wrapped before burial^20^. When soft tissues of the body decay faster than the wrapping, it can create either temporary or semi-permanent spaces around the putrefied body, yielding some skeletal movements at the disarticulated joints before wrapping decay (i.e. right elbow in individual syr013, left shoulder in individual syr005). These individuals were located very close to each other. However, intensive archaeological fieldwork has not yielded evidence of further burials from this period at the site. Therefore, the archaeological record does not support that these burials belong to a cemetery for a specific community. Permits for archaeological samples processed in this study were obtained from the General Directorate of Antiquities and Museums (DGAM), the relevant authority of the Arab Republic of Syria. The remains are curated by DGAM and have been deposited at the Archaeological Museum of As-Suwayda (Syria).

### Radiocarbon dating and Isotopic analysis

Collagen was extracted from two petrous bones following a modified Longin method^79,80^ at the Molecular Archaeology Laboratory at La Trobe University using cold 0.6 M HCl and yields are recorded in Table S14. The collagen was directly radiocarbon dated (AMS) at Waikato University in New Zealand and calibrated using the Oxcal 4.3 programme^81^, and the IntCal13 calibration curve^82^. Radiocarbon results for individuals syr005 and syr013 were 666–768 Cal AD 2σ (1294 ± 18 Cal BP, Lab code wk-46474), and 665–766 Cal AD 2σ (1302 ± 15 Cal BP, Lab code wk-46475), respectively. The collagen stable isotope values and quality parameters were also measured at Waikato and are presented in Table S14. Compound specific stable isotope analysis of amino acids (Table S15) was carried out at the Molecular Archaeology Laboratory, La Trobe University using LC IRMS following methods similar to those described in ref. ^57^ and ref. ^58^. In brief, 1 mg of collagen was hydrolysed under vacuum for 24 h at 110 °C and the hydrolysate was dried in a rotary vacuum concentrator and frozen until required for analysis. The sample was resolved in Milli-Q water with an internal standard (2-amino isobutyric acid). Instrumental analysis was carried out using a three-phase method similar to that described in ref. ^58^ with 10–15 µg of hydrolysate delivered to the column. Isotope values were calculated relative to standard CO_2_ gas peaks delivered throughout the analytical run, with the CO_2_ calibrated against USGS-40.

### Sample preparation and sequencing

Prior to DNA extraction, the petrous bones were UV irradiated (6 J/cm2 at 254 nm) and the first millimetre of bone surface abraded using a Dremel™ tool. DNA was extracted from a 100–200 mg piece of the bone using a silica binding method^83^, with an incubation of 24–48 h, using the MinElute column Zymo extender assembly replaced by the High Pure Extender Assembly (Roche High Pure Viral Nucleic Acid Large Vol. Kit) and performed twice for each sample. Further, blunt-end Illumina multiplex sequencing libraries were prepared^84^ resulting in two double-stranded libraries per sample. Library amplifications were performed as in^85^ using indexed primers^84^ and 4−11 cycles (11 and 10 cycles for syr005; 4 and 6 cycles for syr013). All extraction and library preparation steps were conducted at the dedicated ancient DNA facility at Stockholm University. A total of four DNA libraries were shotgun sequenced on a HiSeq X10 sequencing platform (150 bp paired-end reads) at the NGI Stockholm.

### NGS data processing

Sequenced reads were mapped to the human reference genome build 37 (hs37d5) using *BWA aln*^86^ with non-default parameters −l 16500 −n 0.01 −o 2. Data were merged at the library level using *samtools* v1.5^87^. PCR duplicates were collapsed using a modified version of FilterUniqSAMCons_cc.py^44,88^. Different libraries for one individual were then merged into one bam file using *samtools*v1.5^87^. Reads shorter than 35 bp, showing more than 10% mismatch with the reference and/or a mapping quality score below 30 were discarded. Biological sex was inferred using two different approaches^29,30^.

### Contamination estimates

Contamination for the two individuals  was assessed on three different levels: mitochondria, X-chromosome and autosomes. Two methods were used to estimate mitochondrial contamination^25,26^. X-chromosomal contamination was estimated for the male individual syr005 using the approach implemented in *ANGSD*^28^. *VerifyBAMID*^27^ was used to estimate autosomal contamination in both samples. It uses a hypothetical ‘true genotype’ model and checks whether reads in a bam file are more likely to match a single individual or result from a mixture of other samples, such as a closely related/a different individual.

### Uniparental haplogroups

Mitochondrial haplogroups were identified using Haplofind^89^ and HaploGrep^90^, two web-based applications that use the Human Phylogenetic Tree^91^ to identify the Y-haplotype of sample syr005, reads mapping to the Y chromosome with minimum base and mapping quality of 30 were compared to biallelic substitution SNPs from the International Society of Genetic Genealogy (ISOGG, https://isogg.org/). We excluded A/T and G/C SNPs to avoid strand misidentification and C/T and A/G SNPs to avoid post-mortem damages.

### Population genetic analyses

For population genetic analyses, we merged the ancient samples with modern genotype data of the Human Origins 2.0 dataset^1^ as well as 272 published ancient individuals from the Levant, Europe, Caucasus, North and sub-Saharan Africa^1,6–10,12,39,40,92–98^, resulting in a total of 2854 individuals and 541300 SNPs that passed quality control filters from the Human Origins dataset. We generated pseudohaploid representations of the ancient individuals by randomly drawing one allele from the samtools mpileup^87^ output at each SNP site, restricting the analysis to minimum mapping and read qualities of 30 and coded transition sites as missing to avoid post-mortem damage.

### Principal components analysis (PCA)

A principal component analysis was conducted using *smartpca*^99^ using the options lsqproj and shrinkmode. The first PCA included 73 modern populations from North, West, South, Central and East Europe, Caucasus, Turkey and the Middle East, North and North-east Africa, the Arabian Peninsula, along with 262 published ancient samples from the Levant, North and sub-Saharan Africa and the two ancient UEB individuals (*N* = 1321) from this study. In order to identify the closest modern populations to the UEB individuals, we generated a second PCA with an increased resolution by reducing the original set of modern populations. For this second PCA we excluded divergent groups such as Europeans, Somalis and was limited to 37 modern groups in the Middle East, Turkey and Caucasus henceforth the Middle East, along with ancient individuals (Middle Eastern, *N* = 757 panel). PCA plots were generated using GNU R.

### ADMIXTURE

We used *ADMIXTURE*^34^ for model-based clustering analysis. All data were haploidized by randomly picking one allele per individual at each SNP. Next, the dataset was thinned by pruning out SNPs in linkage disequilibrium using *PLINK* v1.9^100^ with a window size of 200 kb, a step-size of 25 and a squared correlation (*r*^2^) threshold of 0.4. Following this step, 59509 of 541300 variants were removed. *ADMIXTURE* was run for ten iterations with different random starting seeds, five-fold cross validation and the number of ancestral populations (K) was varied from 2 to 10. Results of admixture were visualised using Pong^101^. We ran unsupervised analyses to study the assignment of genetic clusters in the larger set of 73 modern populations from Europe, Middle East, Arabian Peninsula, North Africa and Caucasus and the ancient individuals grouped into 28 population labels determined from their respective studies (*N* = 1321).

### qpAdm

We used *qpAdm*^102^ from the *ADMIXTOOLS*^35^ package with the options allsnps: YES, details: YES and summary: YES to run various modelling scenarios for ancient and modern Levantine groups. First, we modelled the UEB individuals, other ancient and modern Levantine groups as a mixture of four basal ancient populations, namely Neolithic Levant, Neolithic Iran, Western European Hunter-Gatherers and Mota (a 4500-year-old individual from Ethiopia^39^) and their subsets. Secondly, we modelled the UEB individuals as a mixture from ancient Levantines more proximate in time (i.e. Iron/Bronze Age and later periods) along with Mota from Africa^39^. Thirdly, we modelled modern Middle Eastern groups as a mixture of the UEB individuals and Mota. Finally, all models that involve UEB individuals were also run by restricting the models to damaged reads. For this, a likelihood-based approach implemented in *PMDtools*^103^ was used, restricting to sequences with a PMD score of at least 3. For all models, we chose 6 outgroups ('right' populations): Mbuti, Sweden Hunter-Gatherers^92^ (SHG), Kostenki14^104^, Ust-Ishim^105^, CHG^95^ and Neolithic Anatolia^92^. We attempted to implement suggested recommendations for *qpAdm*^106^ and tried to minimise combinations of ancient and modern groups as references, as well as to keep the same set of sources and references for all targets.

### *f* and *D* statistics

To detect shared genetic history between the sequenced samples and various Middle East populations, we used *f*_*3*_ and *D* statistics^35,107^. Population genetic summary statistics were calculated using *POPSTATS*^108^ with sub-Saharan Mbuti rainforest hunter-gatherers as an outgroup. Standard errors were estimated using a weighted block jackknife procedure.

### Conditional nucleotide diversity

To estimate genetic diversity in our data, we used conditional nucleotide diversity (CND)^42^. CND is a measure of genetic diversity within a population based on a comparison of nucleotide differences between two individuals. We used transversion sites that are polymorphic in the Human Origins 2.0 SNP array. Standard errors were estimated using a block jackknife procedure.

### Analysis of genetic kinship

Commonly used approaches for estimating a degree of genetic relationship requires some type of reference data to set a baseline for relatedness. This is usually obtained either by using allele frequencies from modern populations or pairs of unrelated individuals from the same population^109^. Neither of these were an option for this study as we are faced with two individuals from a unique population. Instead, we sampled two distinct reads per individual at each SNP site with at least two reads resulting in two pseudohaploid representations for syr005 and syr013. These representations should behave as identical twins ('pseudo-twins') in a pairwise comparison, meaning that they should show half the genetic distance of an unrelated pair from the same population. Hence, we can compare individual distances to distances between syr005 and syr013 to assess their genetic relationship.

### Phenotypic analysis

Given the observed Arabian ancestry in the sequenced samples, we looked at phenotypic variants that might be expected in this region. For instance, lactase persistence (LP) has been described in the Arabian Peninsula^50^. We also looked for common recessive disorders described in Arab populations from the OMIM (Online Mendelian Inheritance in Man) catalogue^110^. Information about SNP positions and the phenotype was taken from dbSNP^111^ and SNPedia^112^. A pileup file was created in samtools from .bam files with a high base quality (*q* > 30) as well as mapping quality (*Q* > 30) and the occurrence of variants known to be involved in LP and other disorders was checked.

### Diploid genotype calls

We used *snpAD* version 0.3.4^36^, a diploid genotype caller dedicated to work with ancient DNA to call diploid genotypes for syr013. Reads were first realigned around indels using *GATK*^113^. The genotype calls were restricted to reads with mapping quality of at least 30 and base quality of at least 30, calculating an error profile for the first and last 5 bp of each read. Downstream analyses were then restricted to positions with a genotype quality of at least 30 and between 5 and 22 reads sequencing depth at each position.

### ROH

Runs of homozygosity was estimated for modern populations and the diploid calls of syr013 using *PLINK*^100^ with parameters -homozyg-density 50, -homozyg-gap 100, -homozyg-kb 500, -homozyg-snp 100, -homozyg-window-het 1, -homozyg-window-snp 100, -homozyg-window-threshold 0.05 and -homozyg-window-missing 25 for the Human Origins dataset. For the SGDP dataset, the analysis was restricted to 6,383,589 sites with a minor allele frequency of at least 0.01 and no missing data after adding syr013. Therefore, -homozyg-window-missing 25 was set to 1 for the SGDP dataset.

### IBD

Segments showing identity-by-descent between the UEB individual syr013 and modern human populations were determined using *Beagle* 4.1^37^. We used Human Origins 2.0 as well as the Simons Genome Diversity Project (SGDP^46^) to compare results between the HO panel and the SGDP data, which was filtered to remove missing sites from syr013. Beagle’s IBD was run using VCF files with the following parameters: ibdtrim = 30 and 150 for the Human Origins and SGDP datasets respectively; ibd = true; and impute = FALSE, for all chromosomes. We selected segments with a LOD score >3 and length >1 cM for both panels.

### Reporting summary

Further information on research design is available in the Nature Research Reporting Summary linked to this article.

## Supplementary information

Supplementary Information

Description of Additional Supplementary Files

Supplementary Data 1

Reporting Summary

## Data Availability

Generated sequence data is available at the European Nucleotide Archive (ENA) under the accession number PRJEB38008. The remains are curated by the General Directorate of Antiquities and Museums (DGAM) of the Arab Republic of Syria. Raw data for the main figures can be found in Data 1.
