# Supplementary material for: Bioarchaeological evidence of one of the earliest Islamic burials in the Levant
Source: Commun Biol. 2022 Jun 7;5:554. doi: 10.1038/s42003-022-03508-4 (PMC9174286; doi:10.1038/s42003-022-03508-4)
Supplement: Supplementary file 1 — Supplementary Information [file 42003_2022_3508_MOESM1_ESM.pdf]

Supplementary Information

Bioarchaeological analysis of one of the earliest Islamic burials in the Levant

Megha Srigyan[1,a], Héctor Bolívar[2,3], Irene Ureña[2], Jonathan Santana[4], Andrew Petersen[5], Eneko Iriarte[6], Emrah Kırdök[7], Nora Bergfeldt[2], Alice Mora[8], Mattias Jakobsson[1], Khaled Abdo[9], Frank Braemer[10], Colin Smith[6,8], Juan José Ibañez[11], Anders Götherström[2], Torsten Günther[1,*,†], Cristina Valdiosera[6,8*,†]

1Human Evolution, Department of Organismal Biology, Uppsala University, Uppsala, Sweden
a Present address: Department of Ecology and Evolutionary Biology, University of California Santa Cruz, CA, U.S.A. 95064
2 Centre for Palaeogenetics, 10691, Stockholm, Sweden
3 Instituto del Patrimonio Cultural de España, 28040. Madrid, Spain
4 Department of Historical Sciences, Universidad de Las Palmas de Gran Canaria, Las Palmas de G.C., E35001, Spain.
5 University of Wales Trinity Saint David, Lampeter, UK
6 Laboratorio de Evolución Humana, Departamento de Historia, Geografía y Comunicación, Universidad de Burgos, 09001. Burgos, Spain
7 Department of Biotechnology, Mersin University, 33343 Mersin, Turkey
8 Dept. Archaeology and History, La Trobe University, Melbourne, VIC 3086, Australia
9 General Directorate of Antiquities and Museums, Arab Republic of Syria
10 Université Côte d'Azur, CNRS, Culture et Environment, Préhistoire Antiquité Moyen Age
11 Archaeology of Social Dynamics, Milà i Fontanals Institution, Spanish National Research Council (CSIC), Barcelona, Spain

† equal contributions
* Corresponding authors: torsten.guenther@ebc.uu.se, cevaldiosera@ubu.es

This supplement contains

Supplementary Note S1
Supplementary Note S2
Supplementary Figures
Supplementary Tables
References in the Supplementary Note

Supplementary Note S1

History of the excavation and subsequent bioarchaeological analysis

Research on human remains can be a controversial or culturally sensitive subject. We have concluded in this research that the individuals excavated from Qarassa could represent one of the earliest Islamic burials in the Levant. The individuals analysed were not excavated from known Islamic contexts, nor are there such contexts in the local area. The original research focus for the Qarassa project was the Neolithic period. The Islamic interpretation was not an a priori assumption that we could have made, but an interpretation of our data that became apparent in the post excavation analysis (i.e. bioarchaeology and dating).

We include the following information regarding the history of this project to help readers understand how this research was conducted and demonstrate how the Islamic interpretation developed after the excavation during the bioarchaeological analysis.

Excavations

The excavations were undertaken as part of a project to study the Neolithic site of Tell Qarassa. This project was carried out by a Spanish team of the National Research Council (CSIC) directed by Juan José Ibáñez during 2009 and 2010. The research was funded by the Spanish Ministry of Science and Culture and fieldwork was performed through a collaboration of a Spanish team and local archaeologists, including Syrian students on all of the field seasons.. The excavation in Tell Qarassa North was part of a wider archaeological research project around the palaeolake of Qarassa, directed by Frank Braemer of the French National Research Council (CNRS). The archaeological research permit for Qarassa was granted by the General Directorate of Antiquities and Museums (DGAM) of the Arab Republic of Syria. A delegate of the DGAM was present during all the archaeological activities, as required by Syrian Heritage laws and the DGAM's procedures. The permission to excavate included all kinds of archaeological remains, including human burials. Our contribution to the development of Syrian archaeology continues through solid collaborations with Syrian researchers organizing meetings on Heritage protection and managing projects for the consolidation of damaged heritage, the last one funded by the Protection Fund of the British Council (see https://www.rafekatuna.org/). This collaboration has successfully resulted in two fully funded Masters in Archaeology for two Syrian students with one of them achieving a PhD degree.

There are no reports of the existence of a Muslim cemetery in the village of Qarassa, or in the area where the excavation of the Neolithic site was carried out and the closest settlement today is a Druze community, which are not self-identified as Muslims. During the excavation, it was evident that the burial pits with the two individuals in this study were intrusive into the Neolithic archaeological levels. However, at the time of excavation the working hypothesis was that the burials corresponded to a Prehistoric chronology, i.e., the Bronze Age, as other funerary

installations and structures of this period are extensively present around the palaeolake. The human remains recovered in Qarassa, as well as the rest of the archaeological material, were deposited at the Archaeological Museum of Sweida (Syria) under the responsibility of the DGAM (as per their regulations).

Bioarchaeological analysis

Official export permits for the samples were obtained from the DGAM authorities. Fourteen bone samples were delivered by Mr Khaled Abdo (an archaeologist working for the DGAM), to Juan José Ibáñez in Beirut (Lebanon). Dr Ibáñez sent the samples to Dr Cristina Valdiosera, for bioarchaeological analysis as part of the investigations of the Neolithisation process in the Middle East. Ancient DNA analyses were performed on all 14 individuals. However, all samples except two, gave negative results due to bad preservation. A common protocol in aDNA studies is to radiocarbon date samples that yield DNA to establish a chronology for the study. Thus, the two positive samples were sent for radiocarbon dating at the Waikato lab (New Zealand). The radiocarbon dates were surprising as they corresponded with Medieval dates and not the expected prehistoric chronology. While this chronology approximates to the Islamic period it is not sufficient evidence on its own with which to conclude that that these two individuals could potentially be Muslims. This was only contemplated following the analysis of the genetic ancestry and dietary composition of the remains.

In light of these findings, the archaeological context of the burials was reassessed. The genetic results, combined with the radiocarbon dates, the dietary analysis and an appropriate historical and archaeological evaluation suggest that these two individuals may indeed be Medieval Muslims interred in a Neolithic site (i.e secondary use of a prehistoric site). As a result, we have informed the DGAM about the conclusion of our research. Khaled Abdo, co-author of this paper and part of the DGAM, is in direct contact with the current DGAM director planning further actions for the fate of these two burials.

Historical Discussion of Tell Qarassa Burials

Muslim Burials

Muslim graves are very diverse yet easily distinguishable from other forms of religious burial. The diversity is mostly concerned with grave markings, traditions around the funeral and the precise form of the grave pit itself. The defining features of Islamic burials are the position of the body, the lack of a wooden coffin and the speed of burial. Despite the fact that much of the focus of Near Eastern Archaeology has been on funerary remains surprisingly few Islamic burial sites have been investigated. Of the excavated sites, few have been published and even fewer have been subjected to analytical tests. The rarity of archaeologically investigated Muslim burials is

largely the result of religious sensitivities and traditional beliefs about the necessity for having a complete body with which to enter the afterlife (for a detailed discussion of the archaeology of Muslim burials see[1]).

Although death and burial are processes which are nearly always connected with religious belief the Quran has very little to say either about funerals or how a body should be buried (see Quran 5:31, 9:84 and 21:35). Instead, almost all aspects of Muslim practice around death and burial are derived from traditions (*hadith*) and religious reasoning (*ijtihad*). *Hadith* (or traditions) are sayings or actions which are traditionally believed to have come from the Prophet Muhammad. As the example of a perfect Muslim, Muhammad's actions are necessarily in accordance with Islam. Because Muhammad himself did not write down his thoughts or pronouncements, most of the traditions originate from one or other of his close associates who transmitted them orally until such time as they were written. Later the various traditions were collated and organized into four schools (*madhab*) based on their original source and form of transmission. The actual difference between schools is quite small and collectively the different traditions form a body or religious knowledge known as the *Sunnah* (compilation). However, the hadith themselves give limited information about funerary rites and what to do with the body of the deceased and for detailed guidance, Muslim scholars have turned to religious reasoning (*ijtihad*) from which they derive rules based on both the Quran and the traditions[2]. During the thirteenth and fourteenth centuries much of this information was collected in funerary manuals which give specific information on how a dead person should be prepared for funeral and buried[3].

The basic requirements of a Muslim burial as described in Islamic texts are:

1) the deceased should be buried before nightfall on the day of death and if the death occurs too late in the day or at night the burial should take place before sunset the next day.

2) the body should be washed prior to burial and this should usually be carried out by close relatives of the same sex,

3) there is no need for a coffin (although corpses are sometimes carried to the grave within a coffin which is then retrieved for re-use) and the dead should be buried either wrapped in a shroud or where circumstances do not permit in their own clothes.

4) the body should be laid in the grave, usually on their right side, with their head turned to face Mecca. The body was aligned perpendicular to the *Qibla* axis so that only the head faces Mecca. In practical terms this means that in Syria and the Levant (Jordan, Lebanon, Palestine/Israel) the body will be aligned East-West with their face turned towards the south.

5) one of the defining features of Muslim burials is the rule of only one person per grave. This means that husbands and wives are not buried together, and also collective family tombs are forbidden. Very occasionally and in extreme circumstances this can be relaxed for victims of plague or warfare.

According to Muslim tradition, burials and cemeteries should be located outside the inhabited areas usually on the outskirts of a town or village. Visitation of graves by relatives or others is discouraged particularly in fundamentalist Sunni Islam although it is widely practiced and in some cases is an established tradition.

The shape of the burial pit varies, although two main types were commonly used – a single rectangular trench dug out of the ground with a smaller human sized trench at the bottom (*shiqq or shaqq*) or a rectangular trench with a niche in one side where the body is deposited (*lahd*). Another grave form used occasionally is the shaft tomb comprising an underground chamber linked by a tunnel either directly to the surface or via a shallow pit. It seems likely that the different forms of burial originated either at different times and/or in different places.

There is currently little information on this and the small number of archaeologically excavated Muslim burials is too small to draw any conclusions. However, from the very few excavations of early Islamic (pre-ninth century) graves carried out so far, it is evident that simple rectangular burials were the earliest form used with examples uncovered in France[4], Spain[5] and Palestine/Israel[6,7]. Shaft tombs have also been attested from the early eighth century at the site of Nahal 'Oded in the Negev[8]. The earliest archaeologically investigated example of a grave with a side niche (lahad) is from Çatal Höyük in central Anatolia and is dated to the Seljuk period (approx. 11th-13th centuries) or later[9,10].

Archaeology of Muslim Burials

Whilst the number of scientifically excavated Muslim graves is small, the number of early Islamic graves is even less. For example, archaeologists excavating the early Islamic palace at Qastal in Jordan discovered a graveyard dating to the early Umayyad period (late seventh or early eighth century AD) which is of considerable significance for understanding early Islamic society. However, following religious concerns it was not excavated[11]. In most cases the anthropological investigation of early Islamic human remains is only carried out when they are found unexpectedly such as the individuals excavated at Qasr Hallabat who appear to have been murdered[12]. In non-Muslim majority countries, there is usually less reluctance to excavate graves thus the largest number of archaeologically documented Muslim graves comes from the Iberian Peninsula and to a lesser extent from Israel. In Spain and Portugal where the Muslim presence ended more than 500 years ago, a large number of Islamic cemeteries have been excavated. In addition, a wide range of analytical methods have been employed including isotope analysis to determine diet and geographical origins[5], measurement of physical characteristics to investigate ethnic identity[13], and taphonomic investigations to understand post-depositional preservation or remains[14]. In Israel, excavations at the early Islamic city of Ramla uncovered many early Islamic graves including a large cemetery that was subsequently built-over in the ninth century[6,7,15]. The sandy nature of the soil in Ramla meant that the sides of the rectangular graves were lined with stone walls[7]. There has been some anthropological investigation of the skeletons discovered in Ramla although this is rare[16]. Political sensitivities about human remains mean that for most

early Islamic burials discovered in Israel, palaeo-anthropological studies have been limited to recording the position of bodies and the presence or absence of bones.

Early Islamic Southern Syria

Although there had been previous Arab incursions into Syria, the final Muslim conquest of Syria took place in 630's with the capture of Bosra in 634 and Damascus in 636[17]. In 661, Muawiyya, the governor of Syria succeeded to the caliphate and became the first of a series of Umayyad caliphs to rule the Islamic world from Syria. During this period, Damascus was established as the capital and cities throughout Syria and Palestine were developed as centres of Umayyad rule. Also, during this time, the Umayyads and their clients built a series of palaces along the desert fringes of Syria and Jordan[18]. Bosra was the de-facto capital of southern Syria and in the ninth century Ya'qubi described it as the capital of the Hauran as an important area for wheat cultivation[19]. Other important cities in the region included Suwayda, Ezra, Dera and Shehaba.

Tell Qarassa lies at the southern edge of the *Leja,* a forbidding basalt covered region located between the ancient cities of Damascus and Bosra. Although the village of Qarassa appears to be a relatively recent settlement there is evidence of Roman, Byzantine and Medieval occupation both in the immediate area and in the wider region. Archaeological survey work in the region indicates that there was substantial population during the Roman, Byzantine and early Islamic periods with a significant break at the end of the Umayyad period (circa 750). Large scale occupation of the area appears to have resumed in the eleventh and twelfth centuries and continued into the early Ottoman period[20]. Sixteenth century Ottoman tax registers indicate that the Leja region was occupied both by villages and nomadic groups including Turcoman and various Arab Bedouin tribes[21]. The first Druze populations arrived in the region in the seventeenth century fleeing persecution in Mount Lebanon.

The closest historical settlements to Tell Qarassa are Busra al-Harir (6km to the west), Najran (3.5km to the east) and Harran (6km to the north) each of which has significant Roman, medieval and later remains. In addition, there is the small settlement of Duwayra which although not ancient was already in existence in the sixteenth century[21].

Busra al-Harir contains 'extensive ancient and massive' ruins with Greek inscriptions[22]. In the sixteenth century Busra al-Harir was an entirely Muslim settlement with a population of 45 households and 35 single men[23]. Najran, however was exclusively Christian up to the end of the seventeenth century when Druze fleeing persecution in Mount Lebanon settled in the village[24] (Firro 1992, 38-39 and Map No.4). The village contains many ruins and ancient buildings dominated by a sixth century church with two towers[25]. The village of Harran also contains extensive early remains, the most significant of which is a bilingual Arabic Greek inscription dated to 568 AD[26].

Bedouin Burials

The Tell Qarassa graves differ from most other excavated examples of early Islamic burials because they were not located in a cemetery and – from the available evidence – do not seem to have been located near a permanent settlement of the period. Based on this information and also taking into account the DNA results it seems possible that the deceased belonged to a nomadic group similar to Bedouin. Whilst Bedouin are sometimes regarded as the autochthonous form of Arab, in practice they are often regarded as marginal and as such may not have qualified for burial in one of the nearly settlements. The Leja region has a long history of occupation by Bedouin nomads and it is known that the area of Tell Qarassa was occupied by Bedouin of the Banu Sarma in the sixteenth century[21].

Because of the long-term continuity of the Bedouin nomadic lifestyle, burial practices of today or the recent past are regarded as comparable with the distant past back to the early years of Islam[27]. There are a number of aspects of the Tell Qarassa burials which can be documented in Bedouin burials both from the recent and more distant past. Important studies discussing Bedouin burial practices include William and Fidelity Lancaster's (1993) study of the Bedouin graves in north-eastern Jordan[28] and Mustafa and Abu Tayeh's (2013) study of Bedouin funerary practices[29] based on comparing western travellers accounts, comments from focus groups and personal observation. Mustafa and Abu Tayeh[29] draw attention to the fact that many nineteenth century European travellers were unaware of the differences between sedentarized and mobile nomadic Bedouin and thus confused many details of village customs with those of the mobile tribes.

One of the most obvious features of the Tell Qarassa burials is that there were just two graves and no communal cemetery. From studies of nomadic Bedouin, it appears that when someone dies, they are buried immediately in a prominent nearby location[29]. There is a concept of tribal burial grounds located at places with particular associations such as the tomb of the founder of a tribe. In this case the tribe concerned will make an effort to bury the deceased in this location and amongst the Bedouin of Sinai there is a tradition of carrying bodies for several days prior to burial. Mustafa and Abu Tayeh[29] also draw attention to the changing dynamics of nomad groups thus individual tribes or tribal groupings will move spatially through time thus a Bedouin tribe resident in Syria today may have been located in Jordan, Saudi Arabia or even Iraq in early Islamic times. In this respect it is worth noting the genetic evidence from Israel cited in the main text[30] which suggests that the Negev Bedouin arrived around 700 AD.

Re-Use of Ancient Sites

The precise choice of burial ground often involves the re-use of an ancient site; famous examples in the Negev include Tell el-Hesi, Arad, Tell Sheva, Tell Malhatta and Arad[31]. In Syria there are also many examples and at Tell Abu Hureyra, a late Islamic cemetery which covered the Neolithic site, was fully excavated[32]. In some cases the re-use of an ancient site can cause problems of interpretation thus some of the burials from the Qumran cemetery in Palestine/Israel which were previously thought to belong to the Essene community of the 3rd century BC to 1st

century AD have been re-interpreted as belonging to Bedouin from the Mamluk (1260-1516 AD) or Ottoman period (1516-1918)[31]. There are a number of reasons for the re-use of ancient sites as burial grounds - some of these are conscious whilst others may be unconscious. Ancient sites, and in particular Tells are usually higher than the surrounding landscape and form both an important focal point and a viewing platform. Other advantages of ancient sites include the fact that they often have readily available stone which can be used to form a cairn on top of the grave both to deter animals from digging up the body and also to mark the position of the grave. The reality of the threat posed by animals digging up recently buried people is proved by finds of human bones within hyena dens in the Judean desert[31,33]. Also, the deceased persons' clothes are sometimes placed on top of the grave and held down with stones[29]. Another practical reason why ancient sites may be chosen as burial locations is that they are not usually used for cultivation or other agricultural activities which may disturb the graves.

There may, however, be fewer practical reasons for the choice of an ancient site as a burial ground. According to Muslim tradition after his death Muhammad was buried within the confines of his house in Medina[34]. This practice is still sometimes carried out by semi-nomadic Bedouin and examples can be found in the Negev today. In any case derelict buildings were often regarded as good burial locations and Johann Burckhardt noted that if a Bedouin camp is located near an abandoned village and one of the members of the tribe dies the preference is for them to be buried in the ruin[35]. Also ruins have a special status within pre-Islamic Arabic poetry which frequently refer to ruins as symbols of loss and the passing of time. Thus, the pre-Islamic poet Imru al-Qais expressed his sorrow in the following words:

> Stop, Oh my friends, let us pause to weep over the remembrance of my beloved.
>
> Here was her abode on the edge of the sandy desert between Dakhool and Howmal.
>
> The traces of her encampment are not wholly obliterated even now.
>
> For when the South wind blows the sand over them the North wind sweeps it away.
>
> (*Mu'allaqat* Imru al-Qais[36]).

In most cases where ruins are re-utilized as burial sites there is a long gap between the domestic use of a location and its re-use as a burial site. This suggests that those responsible for the burial will have no knowledge or connections with the site although when digging a grave they will be aware of the antiquity of the site particularly if there are earlier pre-historic burials present. This question has been discussed in relation to Çatal Höyük where it has been suggested that there is an awareness of the deep past from the almost continuous use of the site as a burial ground from pre-history to the twentieth century[37]. However, this situation is unusual and, in any case, involves sedentarised villagers rather than nomadic Bedouin.

Identity of the Deceased

The close proximity of the two graves would suggest that the two individuals were related but the genetic results do not suggest a close relationship. Given the proximity of the ^{14}C dates and the fact that these are the only non-prehistoric burials at the site, it seems likely that they died either at the same time or shortly one after the other. The absence of trauma to the bones and the young age of the deceased suggests that they may have died from a disease, possibly the Justinian plague which ravaged the Middle East from 541 AD to 749 AD recurring in cycles of nine to twelve years[38,39]. Specifically, the dates of the burials may be linked to the outbreak of 79 AH (698 AD) which was reported in Syria by as-Suyuṭī[39].

The specific identity of the two people buried at Tell Qarassa in the late seventh or early eighth century as revealed by the DNA analysis provides a tantalizing glimpse of early Islamic society in Syria. At present there are no examples of DNA studies from the region which relate to this period and worldwide the only DNA analysis which relates to early Islamic burials is the study of two individuals from the south of France[4].

Within the context of early Islamic and Late Antique Syria discussed above, the two individuals from Tell Qarassa could fit either of the two scenarios suggested by genome sequencing. In the first scenario the individuals are representative of an Arab society already present in Syria before the Muslim conquest whilst the second scenario suggests that both people were the children or grandchildren of a family which had migrated to Syria with the Muslim conquest. Whilst it is not possible to identify which of the two scenarios applies to these individuals this region of southern Syria had direct connections with Arabia and in particular the Hijaz for centuries[40]. In pre-Islamic times these connections were in the form of direct trade between Mecca and the Hauran. We know for example that one of Muhammad's ancestors Hāshīm organized a bi-annual caravan carrying grain from the Hauran to Mecca and that as a young man Muhammad travelled to Bosra as a merchant (Ṭabarī cited in[40]). The direct connection between Mecca and the Hauran was continued in Islamic times with the annual pilgrimage or Hajj. In addition to the passage of pilgrims the Hauran continued to send supplies to Mecca. The nature of material traded from Mecca to Syria has been a matter of academic controversy, however it seems likely that materials from southern Arabia were probably traded via Mecca as was coffee during the sixteenth and seventeenth centuries[41].

We also know from Late Antique inscriptions in Bosra and the Hauran region that there were substantial family connections with Arabia attested in Nabatean and Safaitic names. Maurice Sartre states that the Quraysh from Mecca probably had agents living in Bosra[40]. Turning from genetic to religious identity, the fact that both individuals were buried according to Muslim tradition is very interesting. Firstly, it provides additional evidence for the early adoption of specific Islamic burial rites which were followed even in remote locations. Secondly it is worth pointing out that although the Hauran was incorporated into the territory of Islam (*Dar al-Islam*) from an early date much of the population remained Christian. The enduring strength of

Christianity in the region is proved by the fact that two churches were built several decades after the Muslim conquest one in 652 (Kafr) and one in 668 (‘Orman)[40]. It therefore seems likely that the two individuals buried at Tell Qarassa were not part of the local sedentarised Arab society which was predominantly Christian but were instead Muslims who were far from the nearest Islamic cemetery. Their presence in the area may either have been because they were nomads camping in the area or perhaps, they were pilgrims utilising one of the near-by Hajj (pilgrim) routes which connected the region to Mecca.

Significance

This study provides further insight into the re-use of prehistoric sites as cemeteries by Muslims. This example raises the question of whether the people undertaking the burial were in some way conscious of the deep history of the site or were simply using it as a convenient location. Given the presence of Neolithic graves on the site, it seems probable that those digging the graves in the late seventh or early eighth centuries AD may have been aware of the earlier internments. Did the presence of these earlier graves make it more acceptable for the early Muslims to bury their relatives in this remote area?

The DNA analysis of the two graves from Tell Qarassa is only the second time that genome sequencing has been applied to early Islamic burials and as such is of considerable significance in efforts to understand the human origins of this dynamic cultural period. The first project to utilise DNA evidence focused on three seventh to ninth century Islamic burials from Nimes in France. The analysis showed that all three individuals were males of Berber and/or Arab and Berber origin and as such provided a useful supplement to the patchy historical information about the period and also the first evidence for a Muslim presence in France[4]. Similarly, the present study provides definitive proof of the early presence of Muslim Arabs in the Syrian countryside.

Supplementary Note S2

Metagenomic analysis of the sequence data

We used two different pipelines to screen the sequencing data for DNA fragments originating from different pathogens. Each sequencing library was analyzed separately with these two pipelines. The first pipeline uses a database that contains complete genomes of archaea, bacteria, viruses and fungal species that are curated from NCBI RefSeq database[46]. This database includes 34 strains of *Yersinia pestis* and one representative genome of variola virus. We aligned sequences to this reference database using the Malt tool[47]. Further, we extracted DNA reads that are assigned to several pathogens (including *Y. pestis* and variola virus). Finally, DNA sequences were aligned back to the reference genomes and we calculated genome-wide authentication statistics (this process includes removing duplicates and calculating deamination statistics).

In the second pipeline, we used the microbial NT database (including nearly all available genomes in NCBI database) and the KrakenUniq[48] tool. We then performed a filtering step to discard species that have less than 200 reads and having less than 1000 unique *k*-mers. Further, we extracted DNA sequences associated with a particular species and aligned these sequences to a database that contains human pathogens using bowtie2[49] tool. We built a custom Pathogenome database based on the extensive literature search. Taxid numbers were extracted from the NCBI taxonomy database and sequences associated to each taxid were downloaded and combined in a fasta file. Bowtie2 tool was used to index this database. The current version contains around 400 pathogenic strains curated from:

- Bode Science centre, relevant pathogen list (~200 taxa) https://www.bode-science-center.com/center/relevant-pathogens-from-a-z.html
- European committee of antimicrobial susceptibility test (~180 taxa) https://eucast.org
- Eukaryotic pathogen database resources (~250 taxa) https://veupathdb.org/veupathdb/app
- NAID emerging Infections Diseases/Pathogens from NIH (~150 taxa) https://www.niaid.nih.gov/research/emerging-infectious-diseases-pathogens
- Sequenced genomes from Sanger institute (~230):
    - Protoza https://www.sanger.ac.uk/resources/downloads/protozoa/
    - Viruses: https://www.sanger.ac.uk/resources/downloads/viruses/
    - Bacteria https://www.sanger.ac.uk/resources/downloads/bacteria/
- Virus pathogen resource (~900 taxa): https://www.viprbrc.org
- Wikipedia bacteria list (~50 taxa)

We filtered the KrakenUniq output, based on authentication criteria for ancient pathogens (see below). Finally, we merged different bam files for each sample, and calculated genome wide authentication statistics.

We ran a genome authentication step that involved removing duplicates and calculating edit distance, read length, and deamination rate. To authenticate ancient pathogen sequences, four criteria are commonly used[50]:

- Edit distance: We expect most of the DNA sequences align with 0 edit distance (0 mismatches) with a declining edit distance distribution
- DNA fragmentation: It is expected to have fragmented DNA sequences and most of the DNA sequences should be less than 100 nucleotides in length
- Deamination patterns: It is expected observe C to T and G to A nucleotide transitions towards the 5' end and 3' end of the DNA fragments, respectively
- Read distribution: Mapped reads should be distributed homogeneously across the genome.

The data for both individuals was obtained from petrous bones which are known to provide high proportions of endogenous DNA. In contrast, teeth are the preferred elements for pathogen studies due to high levels of blood stream in the inner chamber of the tooth[50]. Hence, our power to find pathogen sequences that originate from infections for these two individuals might be limited.

Yersinia pestis **and variola virus**

We found 466 DNA reads aligned to *Y. pestis* in the syr013 sample with the first pipeline. After genome authentication, only 17 DNA reads were left, preventing us from obtaining reliable deamination patterns. However, we could not find any DNA sequences associated with the variola virus.

Mycobacterium tuberculosis

We obtained 4,085 DNA reads associated with *Mycobacterium tuberculosis* in the syr005 sample using the first pipeline. After genome authentication, we were left with 1,793 DNA reads. Figure S6 shows the genome authentication plot. Deamination patterns (Figure S7c) show the nucleotide transition rates that are specific to aDNA damage. Nucleotide length distribution also shows the fragmented DNA profile (Figure S7b). However, edit distance patterns (Figure S7a) do not conform to the expectations. We expect to have a declining edit distance pattern with most of the DNA reads having 0 edit distance. Reads are distributed evenly across the genome with around 2.7% genomic breadth of coverage. These edit distance patterns could be due to a wrong reference genome selection. We used several strains of *M. tuberculosis* but we could not obtain the expected edit distance patterns.

Furthermore, we found nearly 4,000 DNA reads in the syr013 sample. However, the genome authentication step did not deliver any significant results in this sample.

Alcaligenes faecalis

This opportunistic environmental bacteria rarely causes bloodstream infections, for example, endocarditis, meningitis, peritonitis, chronic otitis, abscesses, pyelonephritis and bloodstream infections[51].

We found 35,875 DNA sequences associated with *Alcaligenes faecalis* in syr005 using the second pipeline. After the authentication step we obtained 22,445 sequences. All the three authentication criteria were met for this species (Figure S8). We covered 2.6% (breadth of coverage) of the 5 chromosomal assemblies that we include in tour reference database. The low breadth of coverage could be a result of this effect.

Morganella morganii

M. morganii is a rare human pathogen which can cause sepsis, ecthyma, endophthalmitis, chorioamnionitis, urinary tract infections, soft tissue infections, septic arthritis, meningitis and bacteremia[52].

We found 1,276 DNA sequences associated with this species in the syr005 sample using the second pipeline. After the authentication step, we obtained 502 sequences. Genome authentication plots conform to the expectations (Figure S9). However, we could not obtain a decent coverage for this bacterium due to the low number of sequences.

Mycolicibacterium chitae

M.chitae is a mycobacterial species that occurs mostly in the environment[53]. We found 31,803 DNA sequences associated with this species in the syr005 sample using the second pipeline. After the authentication step we obtained 8,408 DNA sequences. While edit distance patterns do not conform to the expectations, length distribution and deamination patterns show fragmentation and deamination (Figure S10). We obtained 3.4% of genomic coverage in this sample.

In syr005, we identified four different bacteria that are opportunistic pathogens. Furthermore, we found some signal of *M. tuberculosis* in this sample which does not fulfill all authentication criteria. The low number of sequences generally limits our ability to confidently confirm a potential infection of syr005 with these pathogens especially considering that they or related species also occur as soil bacteria.

Supplementary Figures

Figure S1. **a.** Plots of read length distribution **b.** Patterns of base misincorporation at fragment termini.

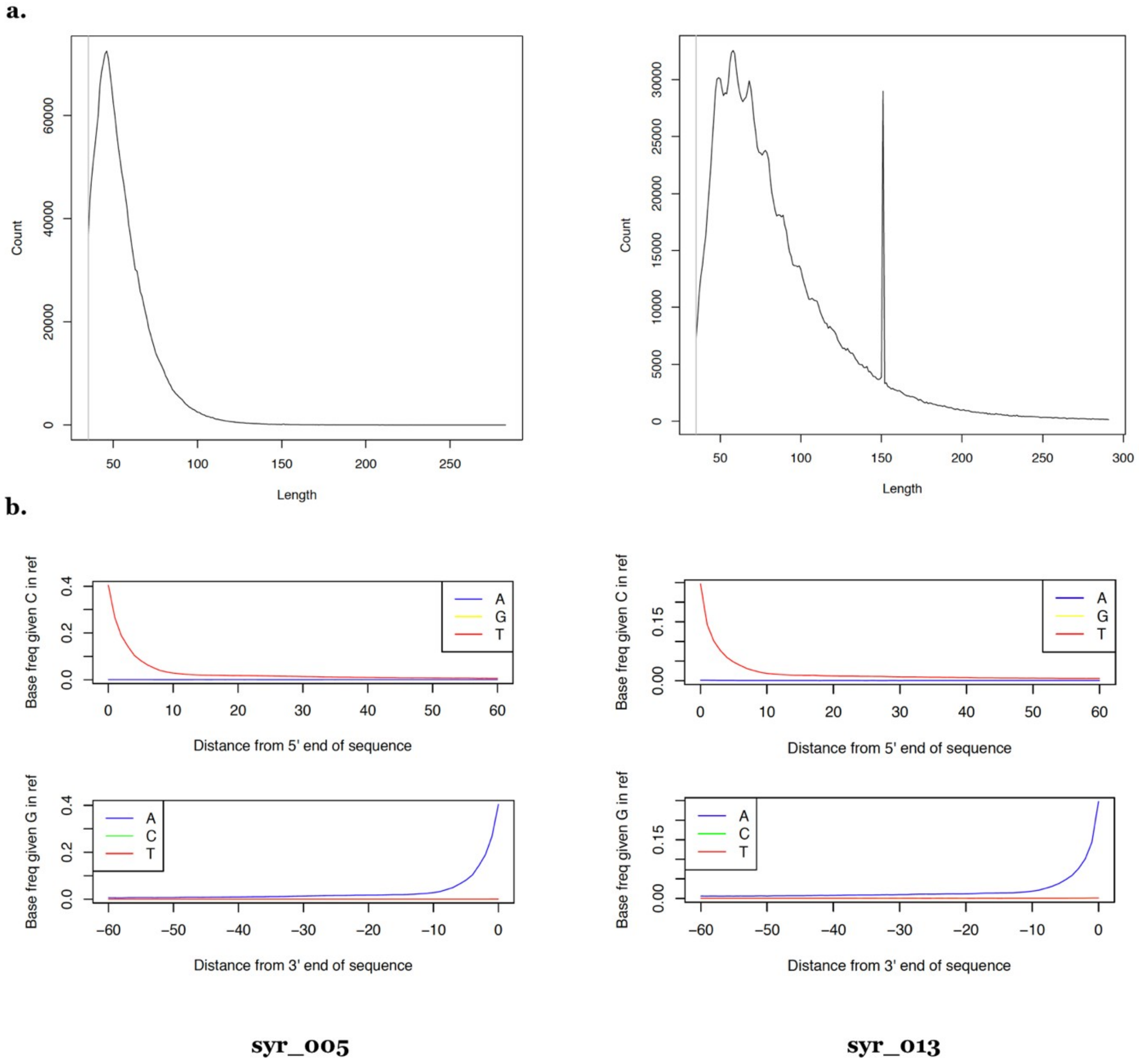

Figure S2. PCA of ancient individuals from the Near East, Africa and UEB individuals (red triangles) projected on modern genetic variation from North Africa, Europe, Middle East, Caucasus and Arabian Peninsula.

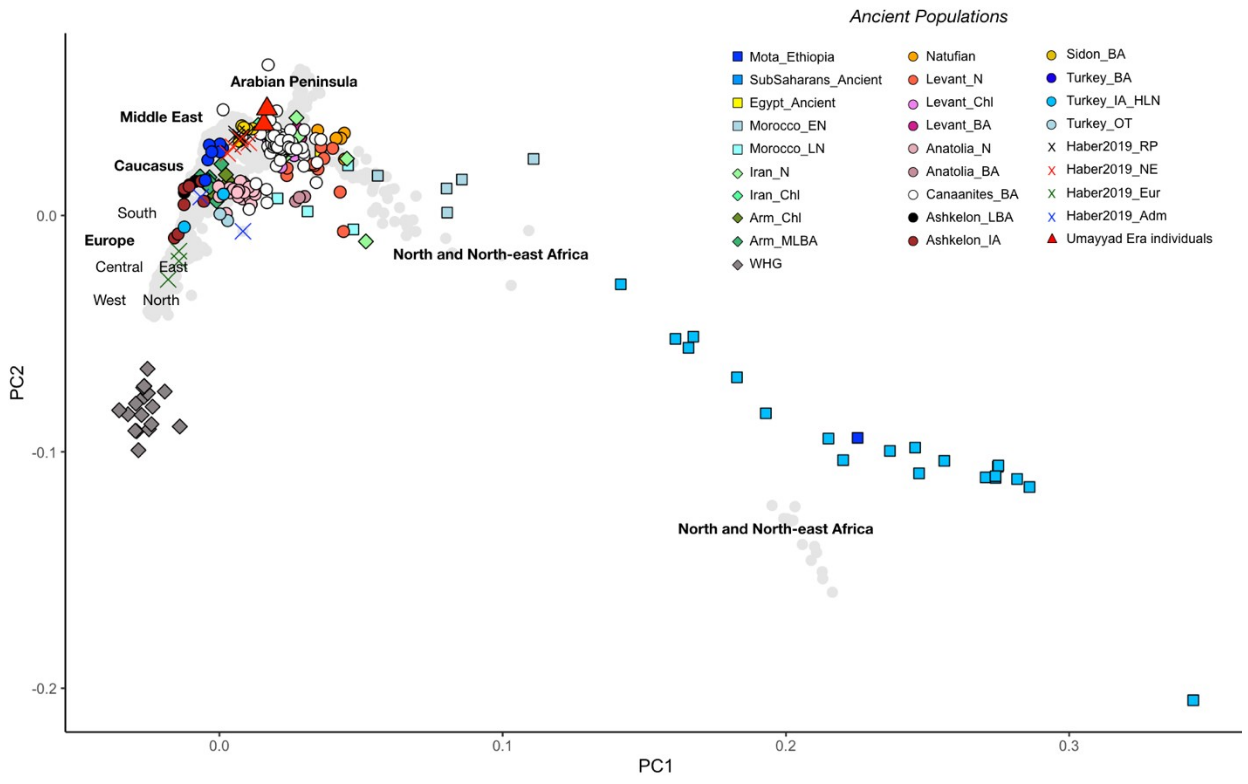

Figure S3. Results of unsupervised ADMIXTURE for K = 2 to K = 10. At K = 2, all other populations are differentiated from ancient African individuals, i.e., Mota from Ethiopia and sub-Saharan Africans [refs]. Groups such as prehistoric Levantines, Neolithic Moroccan individuals and modern Somali groups also showed considerable amount of this component. At K = 3, a new component emerged in WHG and was seen in high amounts in modern European populations.

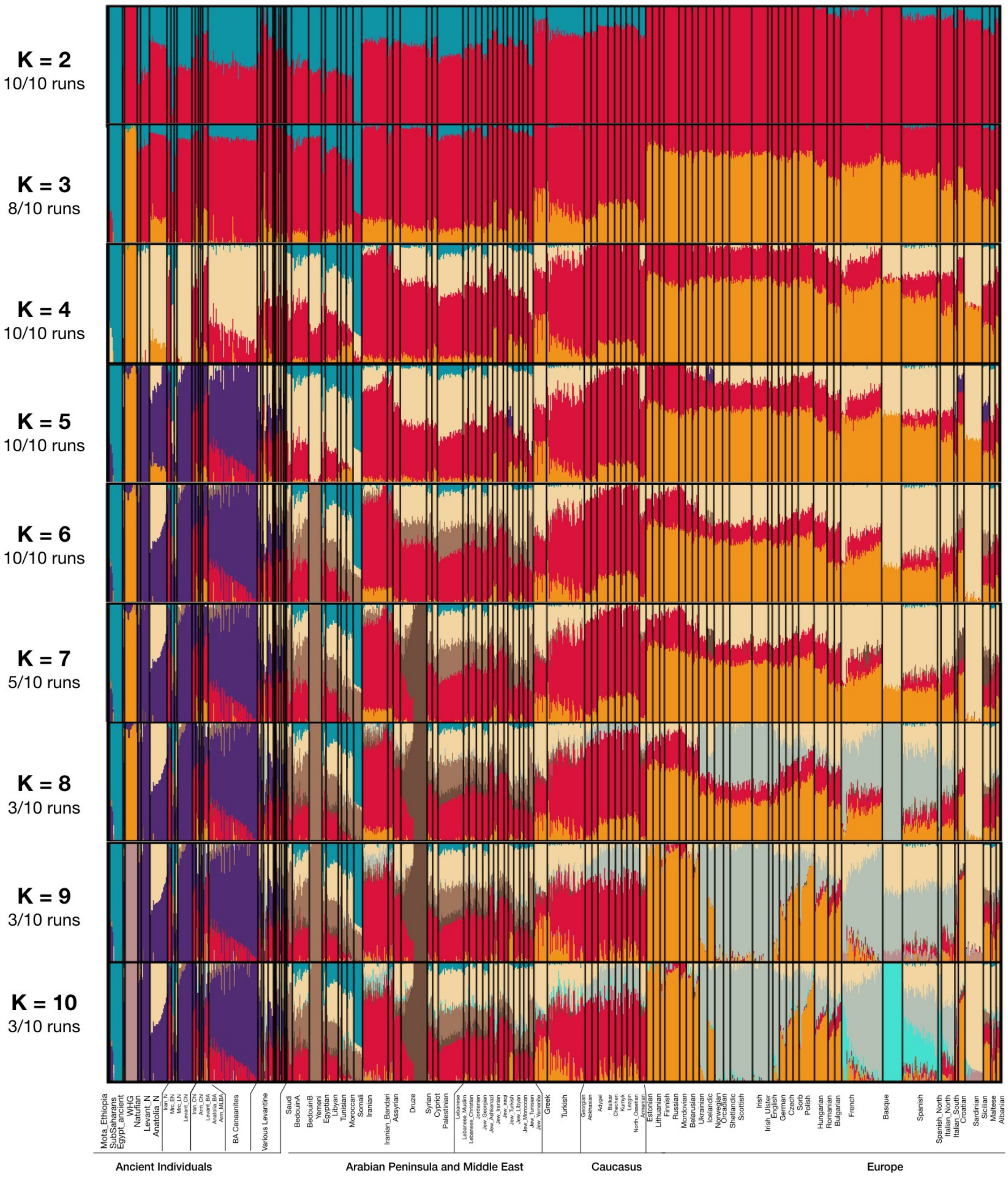
K = 2
10/10 runs
K = 3
8/10 runs
K = 4
10/10 runs
K = 5
10/10 runs
K = 6
10/10 runs
K = 7
5/10 runs
K = 8
3/10 runs
K = 9
3/10 runs
K = 10
3/10 runs
Mota_Ethiopia
SubSaharans
Egypt_ancient
WHG
Natufian
Levant_N
Anatolia_N
BA Canaanites
Various Levantine
Saudi
BedouinA
BedouinB
Yemeni
Egyptian
Libyan
Tunisian
Moroccan
Somali
Iranian
Iranian_Bandari
Assyrian
Druze
Syrian
Cypriot
Palestinian
Greek
Turkish
Estonian
Lithuanian
Finnish
Russian
Mordovian
Belarusian
Ukrainian
Icelandic
Norwegian
Orcadian
Shetlandic
Scottish
Irish
Irish_Ulster
English
German
Czech
Sorb
Polish
Hungarian
Romanian
Bulgarian
French
Basque
Spanish
Spanish_North
Italian_North
Italian_South
Croatian
Sardinian
Sicilian
Maltese
Albanian
Ancient Individuals
Arabian Peninsula and Middle East
Caucasus
Europe

Figure S4. Conditional Nucleotide Diversity (CND) estimates. Standard errors (SE) were calculated using a block-jackknife approach. Error bars represent twice the values of SE.

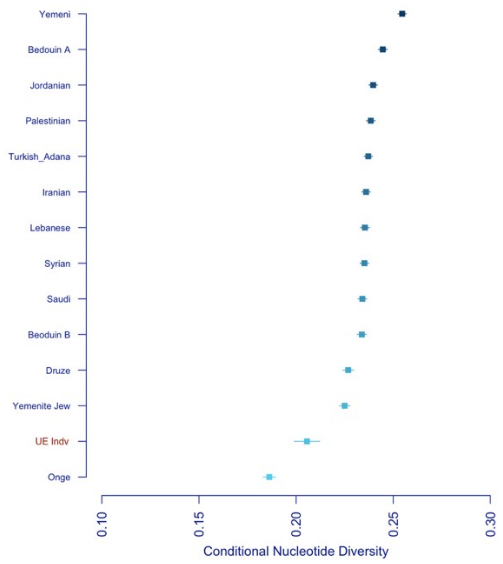

Figure S5. (a) Identity-by-descent (IBD) segments shared between the Umayyad Era Individual syr013 and modern populations and (b) Runs of Homozygosity (ROH) for the UE Individuals using the Simons Genome Diversity Panel (SGDP).

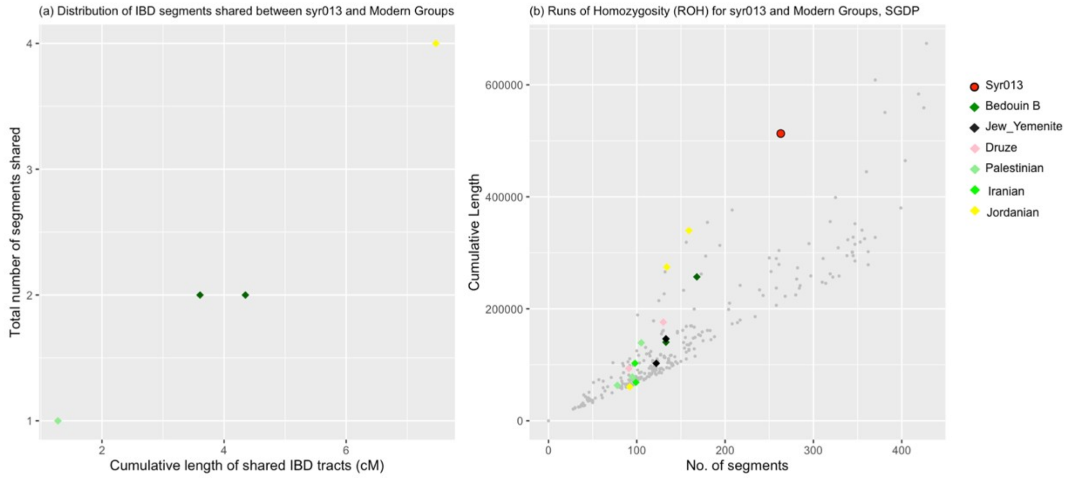

Figure S6: Bulk stable isotope data for Tell Qarassa and other sites in the Levant with temporal proximity.

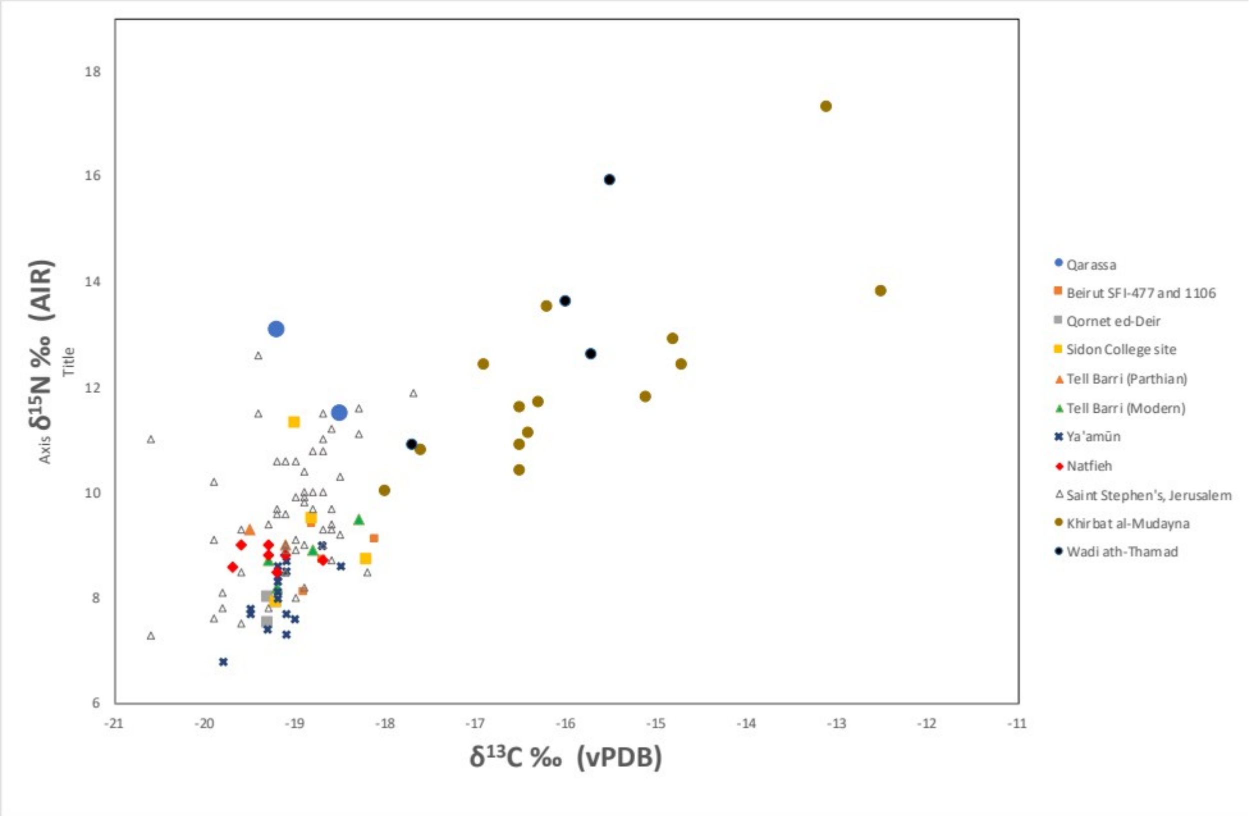

Figure S7: Edit distance **a.**, length distribution **b.**, and deamination patterns **c.** of *Mycobacterium tuberculosis*

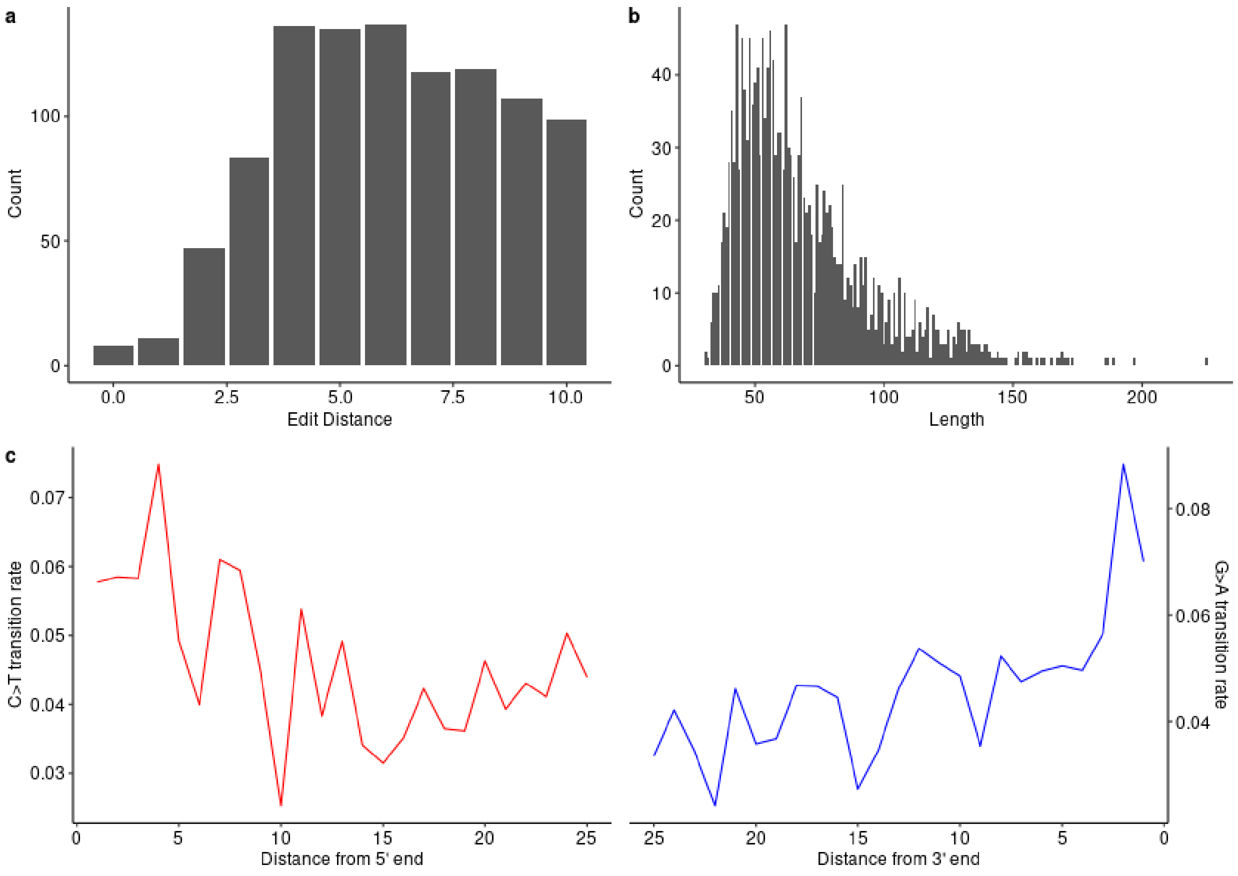

Figure S8: Edit distance **a.**, length distribution **b.**, and deamination patterns **c.** of *Alcaligenes faecalis*.

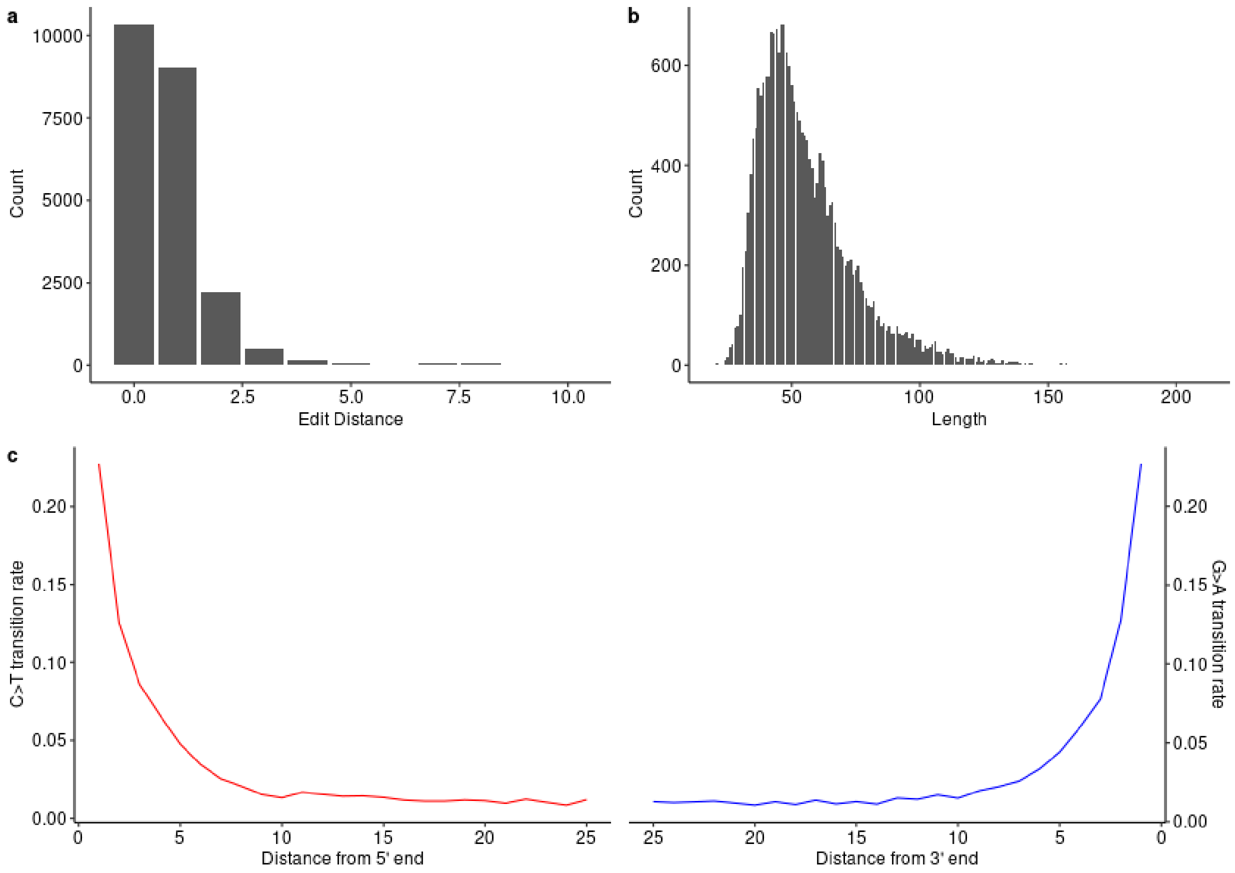

Figure S9: Edit distance **a.**, length distribution **b.**, and deamination patterns **c**. of *Morganella morganii.*

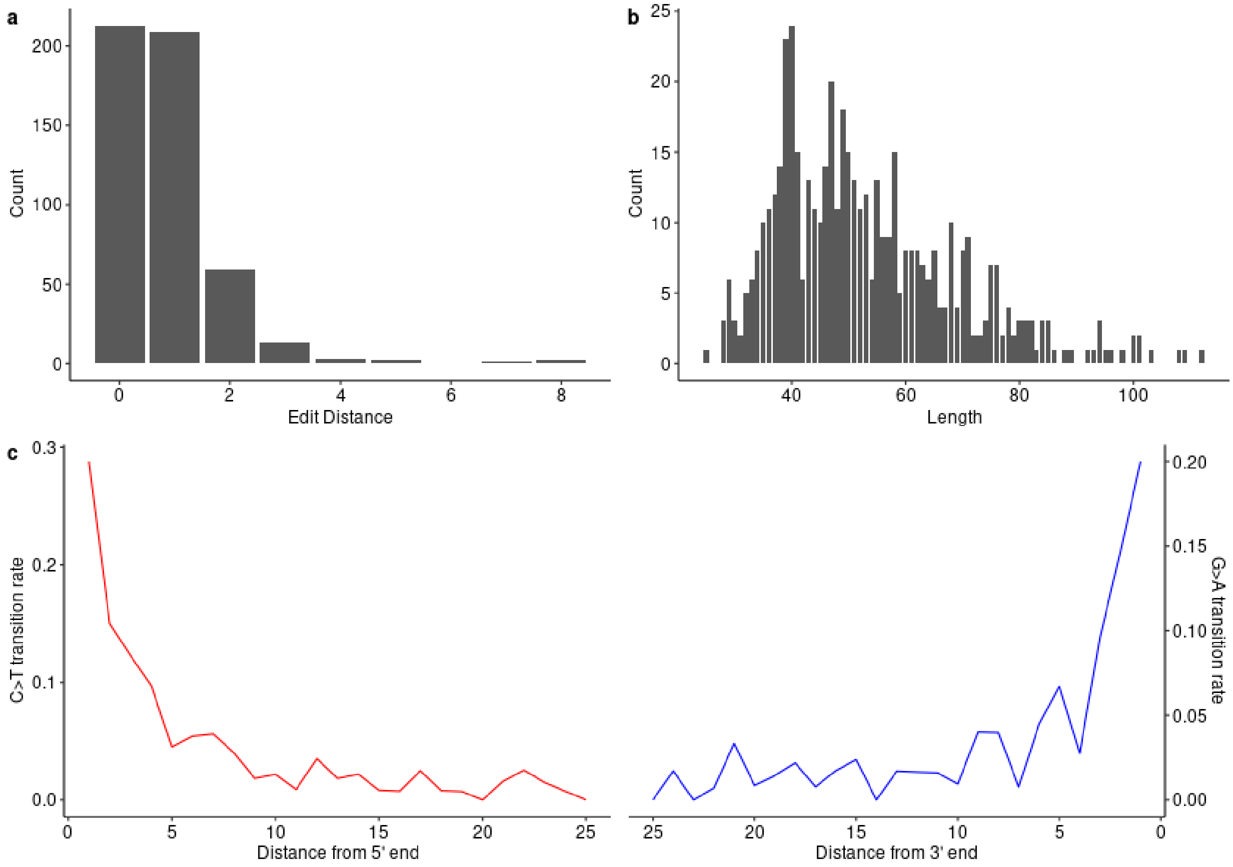

Figure S10: Edit distance **a.**, length distribution **b.**, and deamination patterns **c.** of *Mycolicibacterium chitae.*

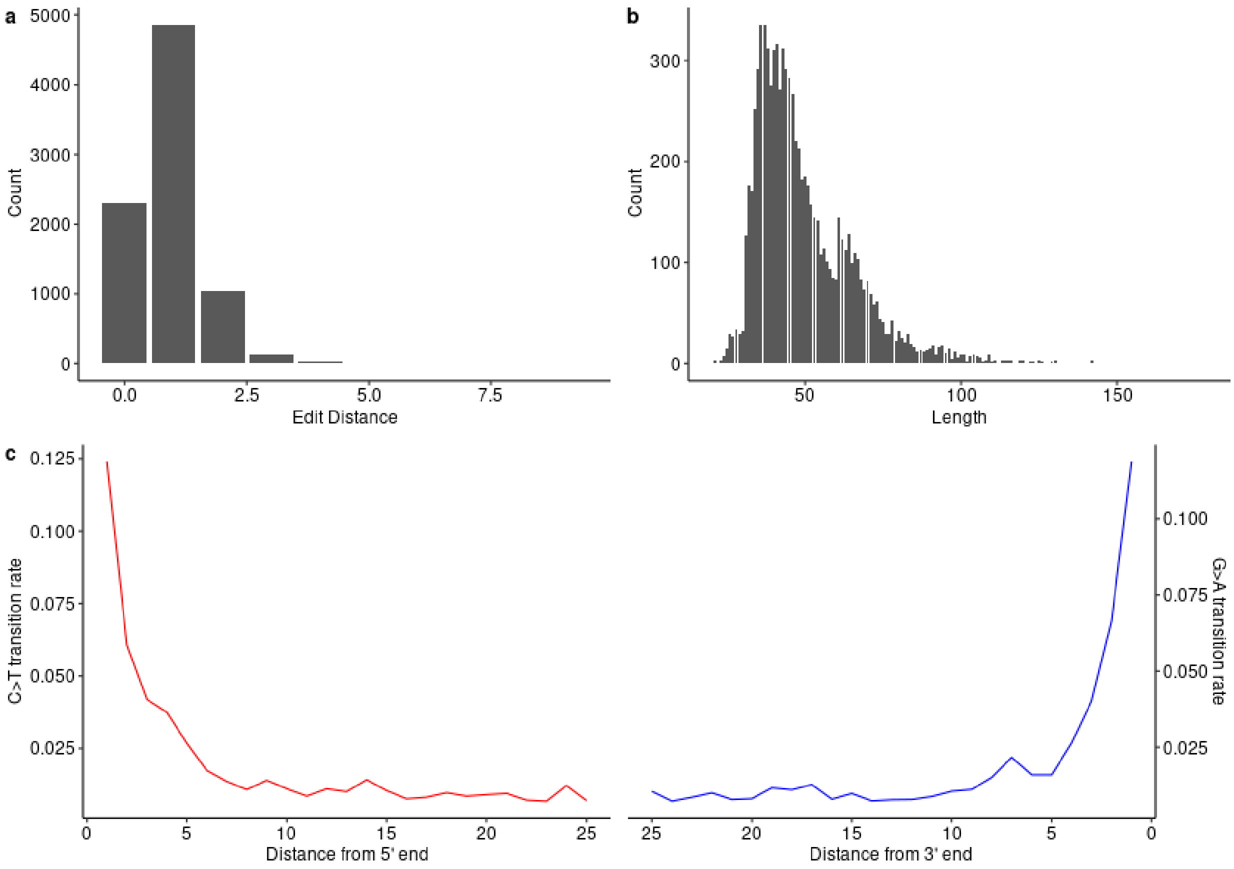

Supplementary Tables

Table S1. Sequencing statistics

Sampl e	Merged reads	Mapped reads (Q30)	Percentage mapped reads	Unique mapped reads	Genome Cov.	Mt Cov
syr005	187836886	12388287	6.59%	9122950	0.159799	23.5158
syr013	537190583	298294035	55.53%	226923995	6.15177	522.349

Table S2. Estimates for mitochondrial, X-chromosomal and autosomal contamination.

Sample	Mt Point estimate	Authentic DNA	X chromosomal	Autosomal
syr005	0	0.9974079	0.027 ± 0.015	0.01475
syr013	0.2	0.9997392	NA	0.0488

Table S3. List of derived Y-chromosome markers for syr005.

Marker	Haplogroup	State
L1013	A1b	derived
PF985	BT	derived
M9129	BT	derived
Z17372	BT	derived
M9344	BT	derived
Z40409	BT	derived
M11760	BT	derived
M5758	CT	derived
Z17706	CT	derived
PF3553	IJ	derived
PF4595	J	derived

Table S4. Values of Outgroup f3 statistics calculated between Syrian samples and Middle East Groups

Population	Outgroup f3 values	Standard Error	Z-score
Abkhasian	0.254594855789	0.00318325880687	79.9793140411
Adygei	0.25261711279	0.00312097736488	80.9416677073
Armenian	0.256306101585	0.00312184922799	82.1007303259
Balkar	0.24991321127	0.00308256981459	81.0730093076
Chechen	0.25409044703	0.00319580365215	79.507527585
Georgian	0.254876365344	0.00316822594601	80.4476605165
Kumyk	0.25061602306	0.00318127729056	78.7784277101
Lezgin	0.25381576998	0.00314674866054	80.6596895273
North_Ossetian	0.251276142262	0.00308113163712	81.5531992318
Jew_Ashkenazi	0.253817916158	0.00323394694435	78.4854917307
Jew_Georgian	0.257439285657	0.0032554989102	79.0782896135
Jew_Iranian	0.255510985398	0.00313014924082	81.6290105489
Jew_iraqi	0.257502031096	0.00322457984111	79.8559948224
Jew_Libyan	0.251014346027	0.00307274610741	81.690558625
Jew_Moroccan	0.250811904783	0.00318326435314	78.7907873675
Jew_Tunisian	0.251031739979	0.00315785663094	79.4943435743
Jew_Turkish	0.253550140602	0.00310015310217	81.7863286897
Jew_Yemenite	0.254212744532	0.00320208510493	79.3897526773
Turkish	0.252926010357	0.00305120903019	82.8937014324

Assyrian	0.256787392083	0.00313905014796	81.8041700449
Syrian	0.2487563738	0.00302800469242	82.1519115947
Cypriot	0.258925502145	0.00318565300986	81.2786268132
Druze	0.254734125606	0.00309133611239	82.4025975645
Jordanian	0.246316466428	0.00306156552561	80.4544160062
Palestinian	0.24762307819	0.00301120610035	82.2338524622
Lebanese_Christian	0.256105828128	0.00309131694565	82.8468360349
Lebanese	0.250572009629	0.00309138325109	81.0549806596
Lebanese_Muslim	0.253559637531	0.00313785442783	80.8066923955
Iranian_Bandari	0.237813908478	0.00302638772186	78.5801193814
Iranian	0.25055504783	0.00305621971934	81.9820140038
**Saudi**	**0.261020315354**	**0.00318728557576**	**81.8942354395**
BedouinA	0.243890411538	0.00296593967958	82.2304017905
**BedouinB**	**0.260777577371**	**0.0030912182945**	**84.3607770552**
Yemeni	0.233549814908	0.00306805239234	76.1231507944
Libyan	0.234981498863	0.00307654572193	76.3783542003
Egyptian	0.235740590839	0.00292750777152	80.5260341687
Sicilian	0.25402133036	0.00309781843235	82.0000706649

Table S5. Top 25 values of D-statistics calculated for the topology (UEB, Bedouin A: X, Mbuti)

**Population X**	**D-Statistic**	**Standard Error**	**Z-score**
BedouinB	0.0564629628856	0.00410478341373	13.7554061188
Saudi	0.0549399728896	0.00409865798404	13.4043809226
Ancient Lebanese	0.0412605758856	0.00425000330273	9.70836325213
Jew_Yemenite	0.0409971343714	0.0041493478683	9.88038016399
Libyan	0.0390960272233	0.00407663947135	9.5902587163
Yemeni	0.038057253172	0.00403666699855	9.42789018408
Jew_iraqi	0.0378467658643	0.00422311149258	8.96182019602
Lebanese	0.0375242122338	0.00393420392806	9.53794285197
Syrian	0.0371670760501	0.00384304726369	9.67125135341
Cypriot	0.0366696596752	0.00411742400429	8.90597121816
Assyrian	0.0364723548016	0.00405615882021	8.99184583697
Moroccan	0.0362499922496	0.00359683978012	10.0782894056
Lebanese_Muslim	0.036164081469	0.00397240201047	9.10383223391
Palestinian	0.035597688354	0.00379190217237	9.38781823366
Jordanian	0.0355131895665	0.00387181472379	9.17223371982
Druze	0.0349766854731	0.00388646113716	8.99962311181
Egyptian	0.0349129648335	0.00371808945157	9.39002820892
Tunisian	0.0348950173804	0.00375081156456	9.30332456849
Jew_Libyan	0.0345635848521	0.00404495795538	8.5448563949
Jew_Georgian	0.0342782325103	0.0042269529855	8.1094425767
Lebanese_Christian	0.0340240847486	0.00404028181464	8.42121572444
Sardinian	0.0338321471761	0.00406666409122	8.31938572186
Jew_Iranian	0.033565728223	0.00409277533107	8.2012144591
Spanish_North	0.0334249294099	0.00428205976085	7.80580638213

Table S6. Top 25 values of D-statistics calculated for the topology (UEB, Bedouin B: X, Mbuti)

**Population X**	**D-Statistic**	**Standard Error**	**Z-score**
BedouinB	-0.0341610012937	0.0039782336333	-8.58697714677
Ancient Lebanese	0.026291560906	0.00441379000542	5.95668594873
Saudi	0.0261054460408	0.00409937098695	6.36815895023
Jew_iraqi	0.022295642107	0.0042744515017	5.216024114
Assyrian	0.0221829651713	0.00418200179668	5.30438920158
Lebanese	0.0219825434304	0.00400912646252	5.48312547281
Cypriot	0.0217910764549	0.00413966754434	5.2639677514
Moroccan	0.0213703151225	0.00368880322283	5.79329224996
Syrian	0.0208263403396	0.00388480243161	5.36097799213
German	0.0207697146259	0.0042561278658	4.87995550904
Jew_Georgian	0.0202655849564	0.00423592624479	4.78421572645

Spanish_North	0.0202254379988	0.00440115353719	4.59548566708
Sardinian	0.0200991237333	0.00409252836798	4.91117517731
Jordanian	0.0200382229666	0.00391563563429	5.1174891737
Albanian	0.0199370934282	0.00430281978698	4.63349487434
Sorb	0.019936714119	0.00423948875342	4.70262224494
Lezgin	0.0197530964642	0.00410136374135	4.81622643343
Jew_Libyan	0.0197093231216	0.00409031639548	4.81853265516
Maltese	0.0197011520089	0.00417564163284	4.71811370353
Lebanese_Muslim	0.0196879048127	0.00400301053448	4.91827454441
Finnish	0.019646681016	0.00426512937165	4.60635054745
Hungarian	0.0196343047155	0.00406343249462	4.83195051018
Tunisian	0.0195532050442	0.00379217564927	5.15619708912
English	0.019514087367	0.0041218570744	4.73429500703

Table S7. Top 25 values of D-statistics calculated for the topology (UEB, Saudi: X, Mbuti)

Population X	D-Statistic	Standard Error	Z-score
Saudi	-0.0583929348666	0.00405681047637	-14.3938040011
BedouinB	0.0255641389308	0.00424494947752	6.02224810121
Ancient Lebanese	0.01929440332	0.00446912820634	4.31726332949
Moroccan	0.0176625967744	0.00373052650449	4.73461232701
BedouinA	0.0174323214495	0.00388842650291	4.48312998496
Jew_iraqi	0.0158841076468	0.00434472918863	3.65594884218
Cypriot	0.0156608350011	0.00421086226633	3.71915156816
Libyan	0.0156206341263	0.00425416414356	3.67184565503
Jew_Yemenite	0.0151611194623	0.00434006761904	3.49329107126
Assyrian	0.0149357199408	0.00425466316801	3.51043533907
Tunisian	0.0147813862816	0.00395616907018	3.73628781261
Lebanese_Muslim	0.0147723022293	0.00414361377426	3.56507701588
Lebanese	0.0145790673824	0.00406060071479	3.59037206718
German	0.014250387461	0.00430932890888	3.30686929736
Palestinian	0.0140957384175	0.00392958122517	3.58708412165
Jew_Libyan	0.0140370839426	0.00418271911731	3.35597097222
Yemeni	0.0139012880884	0.0042479045251	3.27250483298
Syrian	0.0138193977134	0.00400560963958	3.45001109865
Sardinian	0.0137307871554	0.00417695368034	3.28727302388
Jordanian	0.0135778386864	0.00406052461945	3.34386316029
Egyptian	0.0135603758226	0.00388708016814	3.48857631847
Jew_Georgian	0.0135152209864	0.00431782824441	3.13009694258
Druze	0.0132958064824	0.00403972788262	3.29126289411
Maltese	0.0130947512882	0.00426635656552	3.06930541016

Table S8. Top 25 values of D-statistics calculated for the topology (UEB, Jew_Yemenite: X, Mbuti)

Population X	D-Statistic	Standard Error	Z-score
Jew_Yemenite	-0.0753024146011	0.00410592659086	-18.3399320311
BedouinB	0.0335927527277	0.00424168923221	7.9196638152
Saudi	0.0302876568828	0.00424964696151	7.12709953488
Ancient Lebanese	0.0208475673664	0.00444888240869	4.6860234664
Moroccan	0.0193906723602	0.00366894165063	5.2850860566
Libyan	0.0190751952451	0.00424595734584	4.49255460932
BedouinA	0.0183528406453	0.00382403887591	4.7993342225
Syrian	0.0171224631778	0.00392302821415	4.3646036284
Tunisian	0.0168021251074	0.00393390958337	4.2711009878
Lebanese	0.0166019772202	0.00407245172731	4.0766541464
Jew_iraqi	0.0155464166948	0.00431478166375	3.60305987795
Assyrian	0.0154872120216	0.0042554649892	3.63937009489
Jew_Libyan	0.0154399362738	0.00413479798519	3.73414525428
Lebanese_Muslim	0.0152968421969	0.0041097853328	3.72205381988

Yemeni	0.0152780055946	0.00414693654087	3.68416672019
German	0.0151053152765	0.00431762326237	3.49852554486
Jordanian	0.0148170450013	0.00404032788569	3.66728775992
Spanish_North	0.0147878738893	0.00445694575626	3.31793894251
Finnish	0.0147547347438	0.00428253797568	3.4453249049
Egyptian	0.0145873888305	0.00385872929519	3.78036076506
Albanian	0.0144624820943	0.00444636406934	3.25265359939
Druze	0.0143674119715	0.00403675511456	3.55914876275
Icelandic	0.0143296074252	0.00420677077049	3.40632000339
Cypriot	0.014289529261	0.00420532946061	3.39795713864

Table S9. Ancestry proportions for of the Umayyad Era (UEB) Individuals modelled as a 2-way mixture from Ancient NE sources more proximate in time and Mota from Ethiopia. Models were run with full data from the UEB individuals (left) as well as with data restricted to damaged sites (right, UEB Indv_dmg). Working single-source subsets across both types of models are also indicated.

**UEB Individuals**				**UEB Individuals_dmg**				**Working single-sources for both models**
**Ancient NE Source**	**p-value**	**Prop_ANE**	**Mota_Ethiopia**	**Ancient NE Source**	**p-value**	**Prop_ANE**	**Mota_Ethiopia**	
Ash_IA	0.0648	0.98 ± 0.036	0.02 ± 0.036	Ash_IA	0.521	0.997 ± 0.041	0.003 ± 0.041	only Ash_IA works for both
Ash_LBA	0.811853	0.92 ± 0.054	0.080 ± 0.054	Ash_LBA	0.701405	0.861 ± 0.053	0.139 ± 0.053	only Ash_LBA works for both
Sidon_BA	0.339975	0.987 ± 0.015	0.013 ± 0.015	Sidon_BA	0.564753	0.983 ± 0.017	0.017 ± 0.017	only Sidon works for both
AgTam_BA Canaanites (infeasible)	0.00831904	0.98 ± 0.013	0.02 ± 0.013	AgTam_BA Canaanites	0.207156	0.982 ± 0.016	0.018 ± 0.016	only dmg can be modelled just from AgTam_BACanaanites
Haber_2019_NE	0.536474	0.988 ± 0.016	0.012 ± 0.016	Haber_2019_NE	0.923001	0.989 ± 0.018	0.011 ± 0.018	only NE works for both
Haber_2019_RP (infeasible)	0.0382702	0.994 ± 0.015	0.006 ± 0.015	Haber_2019_RP	0.127281	0.991 ± 0.017	0.009 ± 0.017	only RP works for both
Haber_2019_Eur (infeasible)	2.40E-09	0.946 ± 0.018	0.054 ± 0.018	Haber_2019_Eur (infeasible)	4.50E-11	0.94 ± 0.02	0.06 ± 0.02	Nil
Haber_2019_Adm (infeasible)	0.000709646	0.981 ± 0.016	0.019 ± 0.016	Haber_2019_Adm (infeasible)	0.00281408	0.982 ± 0.019	0.018 ± 0.019	Nil
Turkey_BA	0.0891562	0.955 ± 0.014	0.045 ± 0.014	Turkey_BA	0.231819	0.961 ± 0.016	0.039 ±0.016	Nil
Levant_BA (infeasible)	0.004905	0.996 ± 0.017	0.004 ± 0.017	Levant_BA (infeasible)	0.0488283	0.992 ± 0.02	0.008 ± 0.02	Only dmg works with just Levant_BA
Turkey_IA_HLN	0.249717	0.999 ± 0.021	0.001 ± 0.021	Turkey_IA_HLN (infeasible)	0.0597918	1.006 ± 0.025	-0.006 ±0.025	only Turkey_IA_HLN works for both
Turkey_OT (infeasible)	3.57E-09	1.116 ± 0.024	-0.116±0.024	Turkey_OT (infeasible)	4.65E-08	1.129 ± 0.029	-0.129 ±0.029	Nil
Anatolia_BA (infeasible)	0.0648	0.934 ± 0.017	0.066 ± 0.017	Anatolia_BA	1.38E-01	0.935±0.02	0.065±0.02	Nil

Table S10. Ancestry proportions for modern Middle Eastern groups modelled as a 2-way mixture of the Umayyad Era (UEB) Individuals and Mota from Ethiopia. Models were run with full data from the UEB individuals (left) as well as with data restricted to damaged sites (right, UEB Indv_dmg).

Modern Groups from UEB Individuals + Mota_Ethiopia				Models repeated with UEB_dmg			
Target	p-value	UEB Individuals	Mota_Ethiopia	Target	p-value	UEB Indv_dmg	Mota_Ethiopia
BedouinB	0.199072	0.917 ± 0.013	0.083 ± 0.013	BedouinB	0.669068	0.912 ± 0.014	0.088 ± 0.014
Saudi	0.284683	0.933 ± 0.013	0.067 ± 0.013	Saudi	0.524505	0.928 ± 0.015	0.072 ± 0.015
Jew_Yemenite	0.0867404	0.930 ± 0.013	0.070 ± 0.013	Jew_Yemenite	0.17868	0.927 ± 0.015	0.073 ± 0.015
Yemeni	0.631578	0.801 ± 0.013	0.199 ± 0.013	Yemeni	0.419095	0.799 ± 0.014	0.201 ± 0.014
BedouinA	0.35262	0.872 ± 0.012	0.128 ± 0.012	BedouinA	0.659978	0.870 ± 0.014	0.130 ± 0.014
Syrian	0.328197	0.915 ± 0.013	0.085 ± 0.013	Syrian	0.0974721	0.913 ± 0.015	0.087 ± 0.015
Lebanese	0.319278	0.929 ± 0.013	0.071 ± 0.013	Lebanese	0.271321	0.929 ± 0.015	0.071 ± 0.015
Lebanese_Christian	0.193052	0.981 ± 0.014	0.019 ± 0.014	Lebanese_Christian	0.28747	0.980 ± 0.016	0.020 ± 0.016
Lebanese_Muslim	0.349861	0.958 ± 0.013	0.042 ± 0.013	Lebanese_Muslim	0.285665	0.957 ± 0.016	0.043 ± 0.016
Jordanian	0.505962	0.902 ± 0.013	0.098 ± 0.013	Jordanian	0.528545	0.902 ± 0.015	0.098 ± 0.015
Druze	0.175533	0.972 ± 0.013	0.028 ± 0.013	Druze	0.190788	0.971 ± 0.015	0.029 ± 0.015
Cypriot	0.14942	0.999 ± 0.014	0.001 ± 0.014	Cypriot	0.288932	0.999 ± 0.016	0.001 ± 0.016
Palestinian	0.31027	0.914 ± 0.012	0.086 ± 0.012	Palestinian	0.48279	0.913 ± 0.014	0.087 ± 0.014

Table S11. Values of Conditional Nucleotide Diversity calculated for various Middle East groups.The UEB individuals (syr_005 and syr_013) are highlighted in bold.

Sites Differing	Sites Covered	CND Estimate	Standard Error	Samples Compared
140044	598861	0.233850593042	0.00121061614884	BedB_607-608
146980	600819	0.244632742973	0.00114811333385	BedA_609-611
143174	597388	0.23966668229	0.00115364667638	Jordan_214-307
142137	596114	0.238439291813	0.00116059735487	Palestinian_675-676
140107	598468	0.234109426068	0.00113215608327	Saudi_A5-A6
141431	599294	0.235996021986	0.0011065770667	Iran_11-14
134216	596507	0.225003227121	0.00140128791805	Yemenite_Jew_4684-95
135894	599073	0.226840468524	0.00140013283536	Druze_557-558
141112	599423	0.235413055555	0.00117347124191	Lebanon_1-2
142524	600990	0.237148704637	0.00111169782973	TurkAdana_23108-112
109298	586917	0.186223946486	0.00159632020817	Onge1-12
**4024**	**19571**	**0.205610341832**	**0.0032747874575**	**Syr_005-013**
152487	598901	0.254611363147	0.00110476097775	Yemen2-Yemen3
140958	99437	0.235150649693	0.0011392422505	syria364-syria4

Table S12. Lactase persistence variants tested in the samples. The number of reads mapping in syr013 are shown, as well as the alleles seen at the variant site in the reads. For the variant rs41380347 (bold), five derived alleles mapped at this position in syr013.

SNP ID	Chr	Position (GRCh37)	Ancestral	Mutation	Referred to as	No. of Reads Mapped	Alleles Observed	Region of Prevalence	Remarks
rs4988235	2	136608646	G	G > A; G > C	-13,910*T	10	GAGGGGGGGG	Europe	Allele A associated. Also found in some populations from Africa, South Asia
**rs41380347**	2	136608651	A	A > C; A > G	-13,915*G	9	AACCCCCAA	Arabian Peninsula	Allele C associated. East and North Africa, Middle East
rs145946881	2	136608746	C	C > T; C > G	-14,010*C	10	CCCCCCCCCC	Africa	Allele G associated. Identified in East Africa
rs41525747	2	136608643	G	G > C	-13,907*G	10	GGGGGGGGGG	Africa	Allele C associated. Sudan and Ethiopia
rs182549	2	136616754	C	C > T	G/A*14107	11	CCCCCCCCCCC	Africa	Allele T associated. Rare variant, Xhosa

Table S13. Phenotypic variants tested in sample syr013. The clinical significance (as described in dbSNP) of the variant alleles is shown, along with additional remarks about the inheritance/region of prevalence.

Condition	SNP ID	Chr	Position (GRCh37)	Ancestral	Mutation	Reads Mapping	Reads	Remarks
Familial hypercholesterolemia	rs5742904	2	21229160	C	C > A, C > T	8	CCCCCCCC	Allele A pathogenic; T likely pathogenic. Autosomal Dominant
G6PDH Deficiency	rs5030868	X	153762634	G	G > A	5	GGGGG	Other/Likely pathogenic.
G6PDH Deficiency	rs1050828	X	153764217	C	C > T	6	CCCCCC	Allele T pathogenic. 'Middle-east' variant
Sickle-cell anemia	rs334	11	5248232	T	T > A, C, G	5	TTTTT	Allele A other/pathogenic; G other.
Bardet-Biedl Syndrome	rs28938468	15	73027508	C	C > A, G	6	CCCCCC	Allele A pathogenic. Autosomal Recessive
Bardet-Biedl Syndrome	rs28937875	20	10394008	C	C > T	8	CCCCCCCC	Allele T pathogenic. Pleiotropic Disorder
Bardet-Biedl Syndrome	rs2277598	15	73027478	T	T > C	8	CCCCCCC^FC	Allele C benign/minor contributor
Meckel-Gruber Syndrome	rs386834180	8	94793953	T	T > C	4	TTTT	Allele C likely pathogenic. Autosomal Recessive, pleiotropic and lethal
Osteopetrosis	rs398123011	7	26404195	G	G > A	8	GGGGGGGG	Allele A pathogenic. Autosomal Recessive
Marfan Syndrome	rs113871094	15	48758017	G	G > A	6	GGG$GGG	Allele A pathogenic. Autosomal Dominant
Marfan Syndrome	rs137854468	15	48779593	C	C > T	7	CCCCCCC	Allele T pathogenic. Autosomal Dominant.
Congenital chloride diarrhea	rs386833481	7	107431671	G	G > A, C	7	GGGGGGG	Allele A likely pathogenic; C: uncertain significance. Autosomal Recessive
Hypophosphatemic rickets	rs121908249	6	132211575	A	A > C, G	10	AAAAAAAAAA	Allele C pathogenic. Autosomal recessive, prevalent in Bedouins[54]

Table S14. Collagen stable isotope data for the samples analysed in this paper, with similar data for humans from the Levant from proximate periods.

Sample Code	Site	Location	Collagen yield (%)	C%	N%	C:N	$\delta^{13}C$	$\delta^{15}N$	Age	Date	Ref
Syr 005	Qarassa	Syria	13.6	42.2	15.3	3.2	-19.2	13.1	Late Antique	1294 ± 18 Cal BP	This paper
Syr 013	Qarassa	Syria	17.5	41	14.8	3.2	-18.5	11.5	Late Antique	1302 ± 15 Cal BP	This paper
ERS4542973	Beirut SFI-477	Lebanon					-18.1	9.1	Early Roman	119BCE-27CE	Haber et al. 2020[55,56]
ERS4542977	Beirut SFI-477	Lebanon					-18.9	8.1	Early Roman	176BCE-3CE	Haber et al. 2020[55,56]
ERS4542982	Beirut SFI-1106	Lebanon					-18.7	8.7	Early Roman	48CE-222CE	Haber et al. 2020[55,56]
ERS4542980	Beirut SFI-1106	Lebanon					-18.8	9.4	Early Roman	55BCE-58CE	Haber et al. 2020[55,56]
ERS3189338	Qornet ed-Deir	Lebanon					-19.3	8	Late Roman	237CE-389CE	Haber et al. 2019[42,55]
ERS3189333	Qornet ed-Deir	Lebanon					-19.3	7.5	Late Roman	244CE-400CE	Haber et al. 2019[42,55]
ERS3189335	Qornet ed-Deir	Lebanon					-19.2	7.9	Late Roman	426CE-632CE	Haber et al. 2019[42,55]
ERS3189353	Sidon College site	Lebanon					-18.2	8.7	Medieval	1154CE-1281CE	Haber et al. 2019[42,55]
ERS3189352	Sidon College site	Lebanon					-18.8	9.5	Medieval	1187CE-1266CE	Haber et al. 2019[42,55]
ERS3189350	Sidon College site	Lebanon					-19	11.3	Medieval	1191CE-1283CE	Haber et al. 2019[42,55]
ERS3189355	Sidon College site	Lebanon					-19.2	7.9	Medieval	1219CE-1278CE	Haber et al. 2019[42,55]
TB103	Tell Barri	Syria	0.8			3.4	-19.5	9.3	Parthian	c. 100–300 CE	Sołtysiak & Schutkowski (2015)[57]
TB1573	Tell Barri	Syria	5.8			3.2	-19.1	9	Parthian	c. 100–300 CE	Sołtysiak & Schutkowski (2015)[57]
A	Tell Barri	Syria				3.2	-18.8	8.9	Modern	c. 1850–1950 CE	Sołtysiak & Schutkowski (2015)[57]
B	Tell Barri	Syria				3.2	-18.3	9.5	Modern	c. 1850–1950 CE	Sołtysiak & Schutkowski

											(2015)[57]
C	Tell Barri	Syria				3.2	-19.2	8.2	Modern	c. 1850–1950 CE	Sołtysiak & Schutkowski (2015)[57]
D	Tell Barri	Syria				3.2	-19.3	8.7	Modern	c. 1850–1950 CE	Sołtysiak & Schutkowski (2015)[57]
YMN hb 024	Ya'amūn	Jordan	10	41.4	15.1	3.2	-19.1	8.5	Late Roman	c. 300-700CE	Sandias & Muldner (2015)[58]
YMN hb 025	Ya'amūn	Jordan	10	42.8	15.3	3.3	-18.7	9	Late Roman	c. 300-700CE	Sandias & Muldner (2015)[58]
YMN hb 026	Ya'amūn	Jordan	1.3	40.9	14.1	3.4	-19.5	7.7	Late Roman	c. 300-700CE	Sandias & Muldner (2015)[58]
YMN hb 027*	Ya'amūn	Jordan	17	43	15.5	3.2	-18.5	8.6	Byzantine	c. 300-700CE	Sandias & Muldner (2015)[58]
YMN hb 028	Ya'amūn	Jordan	3.5	38.5	13.5	3.3	-19.1	7.3	Byzantine	c. 300-700CE	Sandias & Muldner (2015)[58]
YMN hb 029	Ya'amūn	Jordan	3.6	39.1	13.6	3.4	-19.3	7.4	Byzantine	c. 300-700CE	Sandias & Muldner (2015)[58]
YMN hb 030	Ya'amūn	Jordan	2.6	35.9	12.5	3.3	-19.2	8	Byzantine	c. 300-700CE	Sandias & Muldner (2015)[58]
YMN hb 031	Ya'amūn	Jordan	3.6	42	14.9	3.3	-19.8	6.8	Byzantine	c. 300-700CE	Sandias & Muldner (2015)[58]
YMN hb 032	Ya'amūn	Jordan	3.3	43.4	15.3	3.3	-19.1	7.7	Byzantine	c. 300-700CE	Sandias & Muldner (2015)[58]
YMN hb 033	Ya'amūn	Jordan	3.7	28.9	9.9	3.4	-19.5	7.8	Byzantine	c. 300-700CE	Sandias & Muldner (2015)[58]
YMN hb 034	Ya'amūn	Jordan	5.8	33	11.7	3.3	-19	7.6	Late Roman/Byzantine	c. 300-700CE	Sandias & Muldner (2015)[58]
YMN hb 035	Ya'amūn	Jordan	7.2	38.3	13.5	3.3	-19.2	8.6	Late Roman/Byzantine	c. 300-700CE	Sandias & Muldner (2015)[58]
YMN hb 038	Ya'amūn	Jordan	6	43	15	3.4	-19.2	8.3	Late Roman/Byzantine	c. 300-700CE	Sandias & Muldner (2015)[58]
YMN hb 039	Ya'amūn	Jordan	4.2	41.6	14.3	3.4	-19.2	8.1	Late Roman/Byzantine	c. 300-700CE	Sandias & Muldner (2015)[58]
YMN hb 043	Ya'amūn	Jordan	6.8	43.4	15.6	3.2	-18.7	9	Late Roman/Byzantine	c. 300-700CE	Sandias & Muldner (2015)[58]
YMN hb 044*	Ya'amūn	Jordan	19.5	43.7	15.7	3.2	-19.2	8.4	Late Roman/Byzantine	c. 300-700CE	Sandias & Muldner (2015)[58]
YMN hb 045	Ya'amūn	Jordan	11.5	42.8	15.1	3.3	-19.1	8.7	Late Roman/Byzantine	c. 300-700CE	Sandias & Muldner (2015)[58]
AA77466	Natfieh	Jordan	9.6	40.1	14.4	3.3	-19.6	9	Early Roman	22-236CE	Al-Bashaireh et al (2010)[59]
AA77467	Natfieh	Jordan	10.2	37.5	13.5	3.2	-19.7	8.6	Early Roman	67-258CE	Al-Bashaireh et al (2010)[59]
AA77468	Natfieh	Jordan	9.6	39.9	14.4	3.2	-18.7	8.7	Early Roman	118BCE-81CE	Al-Bashaireh et al (2010)[59]
AA77469	Natfieh	Jordan	9.6	29.9	11	3.2	-19.3	9	Early Roman	51BCE-142CE	Al-Bashaireh et al

											(2010)[59]
AA77470	Natfieh	Jordan	9.1	34.9	12.6	3.2	-19.3	8.8	Early Roman	57BCE-135CE	Al-Bashaireh et al (2010)[59]
AA77475	Natfieh	Jordan	6.1	39.7	14.5	3.2	-19.1	8.8	Early Roman	197BCE-29CE	Al-Bashaireh et al (2010)[59]
AA77476	Natfieh	Jordan	10.5	30.4	11.2	3.2	-19.2	8.5	Early Roman	173BCE-67CE	Al-Bashaireh et al (2010)[59]
EBND 6.318	Saint Stephen's, Jerusalem	Israel/Palestine		46.5	15.6	3.5	-19.6	7.5	Byzantine	c400-700CE	Gregoricka & Sheridan (2013)[60]
EBND 3.118	Saint Stephen's, Jerusalem	Israel/Palestine		42.1	13.8	3.6	-19.2	9.6	Byzantine	c400-700CE	Gregoricka & Sheridan (2013)[60]
EBND 22.382	Saint Stephen's, Jerusalem	Israel/Palestine		43.5	14.6	3.5	-18.6	9.7	Byzantine	c400-700CE	Gregoricka & Sheridan (2013)[60]
EBND 12.134	Saint Stephen's, Jerusalem	Israel/Palestine		48.4	16.1	3.5	-18.3	11.6	Byzantine	c400-700CE	Gregoricka & Sheridan (2013)[60]
EBND 12.601	Saint Stephen's, Jerusalem	Israel/Palestine		45.5	15.6	3.4	-20.6	7.3	Byzantine	c400-700CE	Gregoricka & Sheridan (2013)[60]
EBND 6.261	Saint Stephen's, Jerusalem	Israel/Palestine		45.8	15.4	3.5	-19.9	9.1	Byzantine	c400-700CE	Gregoricka & Sheridan (2013)[60]
EBND 1.36	Saint Stephen's, Jerusalem	Israel/Palestine		43.6	14.7	3.5	-19.9	7.6	Byzantine	c400-700CE	Gregoricka & Sheridan (2013)[60]
EBND 4.360	Saint Stephen's, Jerusalem	Israel/Palestine		35.7	11.9	3.5	-19.9	10.2	Byzantine	c400-700CE	Gregoricka & Sheridan (2013)[60]
EBND 11.674	Saint Stephen's, Jerusalem	Israel/Palestine		45.4	15.8	3.4	-19.8	7.8	Byzantine	c400-700CE	Gregoricka & Sheridan (2013)[60]
EBND 1.199	Saint Stephen's, Jerusalem	Israel/Palestine		43.7	14.7	3.5	-19.8	8.1	Byzantine	c400-700CE	Gregoricka & Sheridan (2013)[60]
EBND 3.154	Saint Stephen's, Jerusalem	Israel/Palestine		45.2	15.1	3.5	-19.6	8.5	Byzantine	c400-700CE	Gregoricka & Sheridan (2013)[60]
EBND 25.239	Saint Stephen's, Jerusalem	Israel/Palestine		42	14	3.5	-19.4	12.6	Byzantine	c400-700CE	Gregoricka & Sheridan (2013)[60]
EBND 5.433	Saint Stephen's, Jerusalem	Israel/Palestine		45.4	15.6	3.4	-19.4	11.5	Byzantine	c400-700CE	Gregoricka & Sheridan (2013)[60]
EBND 1.376	Saint Stephen's,	Israel/Palestine		46.6	15.7	3.5	-19.3	9.4	Byzantine	c400-700CE	Gregoricka & Sheridan (2013)[60]

	Jerusalem										
EBND 1.47	Saint Stephen's, Jerusalem	Israel/Palestine		47	15.3	3.6	-19.2	10.6	Byzantine	c400-700CE	Gregoricka & Sheridan (2013)[60]
EBND 3.144	Saint Stephen's, Jerusalem	Israel/Palestine		46.2	16.1	3.3	-19.1	8.5	Byzantine	c400-700CE	Gregoricka & Sheridan (2013)[60]
EBND 3.127	Saint Stephen's, Jerusalem	Israel/Palestine		47	16.2	3.4	-19.1	8.9	Byzantine	c400-700CE	Gregoricka & Sheridan (2013)[60]
EBND 8.77	Saint Stephen's, Jerusalem	Israel/Palestine		45.3	15.2	3.5	-19.1	9.6	Byzantine	c400-700CE	Gregoricka & Sheridan (2013)[60]
EBND 4.95	Saint Stephen's, Jerusalem	Israel/Palestine		46.2	15.5	3.5	-19.1	8.5	Byzantine	c400-700CE	Gregoricka & Sheridan (2013)[60]
EBND 6.175	Saint Stephen's, Jerusalem	Israel/Palestine		44.3	15.2	3.4	-19	8	Byzantine	c400-700CE	Gregoricka & Sheridan (2013)[60]
EBND 1.146	Saint Stephen's, Jerusalem	Israel/Palestine		43.9	15.5	3.3	-19	8.9	Byzantine	c400-700CE	Gregoricka & Sheridan (2013)[60]
EBND 1.325	Saint Stephen's, Jerusalem	Israel/Palestine		47.3	15.2	3.6	-19	9.1	Byzantine	c400-700CE	Gregoricka & Sheridan (2013)[60]
EBND 9.325	Saint Stephen's, Jerusalem	Israel/Palestine		41.8	13.8	3.5	-18.9	9.9	Byzantine	c400-700CE	Gregoricka & Sheridan (2013)[60]
EBND 2.167	Saint Stephen's, Jerusalem	Israel/Palestine		40.6	13.4	3.6	-18.9	10.4	Byzantine	c400-700CE	Gregoricka & Sheridan (2013)[60]
EBND 4.174	Saint Stephen's, Jerusalem	Israel/Palestine		45.2	15.1	3.5	-18.9	10	Byzantine	c400-700CE	Gregoricka & Sheridan (2013)[60]
EBND 2.282	Saint Stephen's, Jerusalem	Israel/Palestine		39.9	13.8	3.4	-18.9	9.8	Byzantine	c400-700CE	Gregoricka & Sheridan (2013)[60]
EBND 3.117	Saint Stephen's, Jerusalem	Israel/Palestine		47.7	15.6	3.6	-18.8	10.8	Byzantine	c400-700CE	Gregoricka & Sheridan (2013)[60]
EBND 4.169	Saint Stephen's, Jerusalem	Israel/Palestine		38.8	12.9	3.5	-18.8	10	Byzantine	c400-700CE	Gregoricka & Sheridan (2013)[60]
EBND 1.407	Saint Stephen's, Jerusalem	Israel/Palestine		41.1	13.5	3.6	-18.7	9	Byzantine	c400-700CE	Gregoricka & Sheridan (2013)[60]
EBND 22.201	Saint Stephen's,	Israel/Palestine		39.6	13.2	3.5	-18.7	11.5	Byzantine	c400-700CE	Gregoricka & Sheridan (2013)[60]

	Jerusalem										
EBND 1.38	Saint Stephen's, Jerusalem	Israel/Palestine		43.3	14.9	3.4	-18.7	10	Byzantine	c400-700CE	Gregoricka & Sheridan (2013)[60]
EBND 5.163	Saint Stephen's, Jerusalem	Israel/Palestine		45.3	15.4	3.4	-18.7	11	Byzantine	c400-700CE	Gregoricka & Sheridan (2013)[60]
EBND 3.131	Saint Stephen's, Jerusalem	Israel/Palestine		42.2	14.5	3.4	-18.7	10.8	Byzantine	c400-700CE	Gregoricka & Sheridan (2013)[60]
EBND 1.125	Saint Stephen's, Jerusalem	Israel/Palestine		44	14.7	3.5	-18.7	9.3	Byzantine	c400-700CE	Gregoricka & Sheridan (2013)[60]
EBND 2.171	Saint Stephen's, Jerusalem	Israel/Palestine		47.1	16.2	3.4	-18.6	11.2	Byzantine	c400-700CE	Gregoricka & Sheridan (2013)[60]
EBND 6.321	Saint Stephen's, Jerusalem	Israel/Palestine		46	16.2	3.3	-18.6	8.7	Byzantine	c400-700CE	Gregoricka & Sheridan (2013)[60]
EBND 6.122	Saint Stephen's, Jerusalem	Israel/Palestine		44.3	14.8	3.5	-18.5	10.3	Byzantine	c400-700CE	Gregoricka & Sheridan (2013)[60]
EBND 2.166	Saint Stephen's, Jerusalem	Israel/Palestine		42.9	14.8	3.4	-18.5	9.2	Byzantine	c400-700CE	Gregoricka & Sheridan (2013)[60]
EBND 4.186	Saint Stephen's, Jerusalem	Israel/Palestine		47.3	16.6	3.3	-18.2	8.5	Byzantine	c400-700CE	Gregoricka & Sheridan (2013)[60]
EBND 1.33	Saint Stephen's, Jerusalem	Israel/Palestine		41.1	13.5	3.6	-19.3	7.8	Byzantine	c400-700CE	Gregoricka & Sheridan (2013)[60]
EBND 1.358	Saint Stephen's, Jerusalem	Israel/Palestine		46.3	16	3.4	-18.3	11.1	Byzantine	c400-700CE	Gregoricka & Sheridan (2013)[60]
EBND 1.359	Saint Stephen's, Jerusalem	Israel/Palestine		38.4	13.3	3.4	-19.1	10.6	Byzantine	c400-700CE	Gregoricka & Sheridan (2013)[60]
EBND 1.409	Saint Stephen's, Jerusalem	Israel/Palestine		43.9	15.1	3.4	-19	9.9	Byzantine	c400-700CE	Gregoricka & Sheridan (2013)[60]
EBND 2.287	Saint Stephen's, Jerusalem	Israel/Palestine		43.2	14.5	3.5	-19	10.6	Byzantine	c400-700CE	Gregoricka & Sheridan (2013)[60]
EBND 3.114	Saint Stephen's, Jerusalem	Israel/Palestine		44.2	15.4	3.4	-19.6	9.3	Byzantine	c400-700CE	Gregoricka & Sheridan (2013)[60]
EBND 4.206	Saint Stephen's,	Israel/Palestine		46.2	15.5	3.5	-18.6	9.3	Byzantine	c400-700CE	Gregoricka & Sheridan (2013)[60]

	Jerusalem										
EBND 4.4296	Saint Stephen's, Jerusalem	Israel/Palestine		47.7	16.2	3.4	-18.9	9	Byzantine	c400-700CE	Gregoricka & Sheridan (2013)[60]
EBND 6.135	Saint Stephen's, Jerusalem	Israel/Palestine		45.9	15.6	3.4	-18.9	8.2	Byzantine	c400-700CE	Gregoricka & Sheridan (2013)[60]
EBND 6.228	Saint Stephen's, Jerusalem	Israel/Palestine		47	15.3	3.6	-20.6	11	Byzantine	c400-700CE	Gregoricka & Sheridan (2013)[60]
EBND 6.238	Saint Stephen's, Jerusalem	Israel/Palestine		46.8	15.3	3.6	-19.2	9.7	Byzantine	c400-700CE	Gregoricka & Sheridan (2013)[60]
EBND 7.436	Saint Stephen's, Jerusalem	Israel/Palestine		43	14.8	3.4	-18.8	9.7	Byzantine	c400-700CE	Gregoricka & Sheridan (2013)[60]
EBND 10.865	Saint Stephen's, Jerusalem	Israel/Palestine		46.1	15.4	3.5	-17.7	11.9	Byzantine	c400-700CE	Gregoricka & Sheridan (2013)[60]
EBND 21.166	Saint Stephen's, Jerusalem	Israel/Palestine		37.9	12.4	3.6	-18.6	9.4	Byzantine	c400-700CE	Gregoricka & Sheridan (2013)[60]
EBND 27.255	Saint Stephen's, Jerusalem	Israel/Palestine		46.6	15.6	3.5	-19.1	9	Byzantine	c400-700CE	Gregoricka & Sheridan (2013)[60]
G9:6	Khirbat al-Mudayna	Jordan		45.1	16	3.3	-16.5	10.4	Historical Bedouin	c1200-1900CE	Gregoricka & Judd (2016)[61]
N74:17	Khirbat al-Mudayna	Jordan		28.5	9.9	3.4	-18	10	Historical Bedouin	c1200-1900CE	Gregoricka & Judd (2016)[61]
L33:36	Khirbat al-Mudayna	Jordan		46.8	16.6	3.3	-15.1	11.8	Historical Bedouin	c1200-1900CE	Gregoricka & Judd (2016)[61]
N63:12	Khirbat al-Mudayna	Jordan		45.9	16.7	3.2	-12.5	13.8	Historical Bedouin	c1200-1900CE	Gregoricka & Judd (2016)[61]
L13:25	Khirbat al-Mudayna	Jordan		44.6	15.9	3.3	-16.5	10.9	Historical Bedouin	c1200-1900CE	Gregoricka & Judd (2016)[61]
E99:27	Khirbat al-Mudayna	Jordan		46.8	17.3	3.2	-14.8	12.9	Historical Bedouin	c1200-1900CE	Gregoricka & Judd (2016)[61]
N43:27	Khirbat al-Mudayna	Jordan		45.5	15.9	3.3	-16.5	11.6	Historical Bedouin	c1200-1900CE	Gregoricka & Judd (2016)[61]
U29:20	Khirbat al-Mudayna	Jordan		47.8	16.3	3.4	-16.4	11.1	Historical Bedouin	c1200-1900CE	Gregoricka & Judd (2016)[61]
T92:8	Khirbat al-Mudayna	Jordan		46.2	16.8	3.2	-17.6	10.8	Historical Bedouin	c1200-1900CE	Gregoricka & Judd (2016)[61]
112 B16	Wadi ath-Thamad	Jordan		40.1	14.1	3.3	-15.7	12.6	Historical Bedouin	c1200-1900CE	Gregoricka & Judd (2016)[61]
140:3	Wadi ath-Thamad	Jordan		47.8	16.9	3.3	-17.7	10.9	Historical Bedouin	c1200-1900CE	Gregoricka & Judd (2016)[61]
112 B1:39	Wadi ath-	Jordan		47.3	16.8	3.3	-13.6	14.8	Historical	c1200-1900CE	Gregoricka & Judd

	Thamad								Bedouin		(2016)[61]
G9:4	Khirbat al-Mudayna	Jordan		43.3	15.6	3.2	-16.2	13.5	Historical Bedouin	c1200-1900CE	Gregoricka & Judd (2016)[61]
N64:21	Khirbat al-Mudayna	Jordan		45.2	16.4	3.2	-13.1	17.3	Historical Bedouin	c1200-1900CE	Gregoricka & Judd (2016)[61]
A16:10	Khirbat al-Mudayna	Jordan		42.4	15.4	3.2	-16.3	11.7	Historical Bedouin	c1200-1900CE	Gregoricka & Judd (2016)[61]
N54:8	Khirbat al-Mudayna	Jordan		46.3	15.7	3.4	-16.9	12.4	Historical Bedouin	c1200-1900CE	Gregoricka & Judd (2016)[61]
U29:14	Khirbat al-Mudayna	Jordan		45.5	15.7	3.4	-14.7	12.4	Historical Bedouin	c1200-1900CE	Gregoricka & Judd (2016)[61]
112 B2:19	Wadi ath-Thamad	Jordan		44.9	16.6	3.2	-16	13.6	Historical Bedouin	c1200-1900CE	Gregoricka & Judd (2016)[61]
61A:27	Wadi ath-Thamad	Jordan		39.3	13.5	3.4	-15.5	15.9	Historical Bedouin	c1200-1900CE	Gregoricka & Judd (2016)[61]

Table S15. Compound specific carbon stable isotope data for collagen hydrolysate amino acids from Tell Qarassa

**Sample**	**Syr005**		**Syr013**	
**Amino Acid**	**δ13C ‰ (vPDB)**	**S.D.**	**δ13C ‰ (vPDB)**	**S.D.**
**Ala**	-23.5	0.6	-21.5	0
**Arg**	-21.2	0.8	-20.3	0.1
**Asx**	-21.8	0.4	-20	0.1
**Glx**	-16.6	0.2	-15.5	0.1
**Gly**	-17.5	0.5	-15.1	0.1
**His-Ile coeluted**	-26.2	0.6	-24.1	0
**Hyl**	-20.6	0.2	-19.5	0.3
**Hyp**	-19.5	0.6	-19.4	0.2
**Leu**	-30.3	0.6	-30	0
**Lys**	-22.5	0.8	-22.9	0
**Met**	-25.1	0.8	-24.3	0.8
**Phe**	-28.1	0.8	-27.4	0.1
**Pro**	-20.1	0.6	-20	0
**Ser**	-21	0.5	-18.4	0.6
**Thr**	-16.2	0.7	-11.7	0.1
**Val**	-27.3	0.4	-27.2	0
**$\delta^{13}C_{GLY}$-$\delta^{13}C_{PHE}$**	10.6	N/A	12.3	N/A
**$\delta^{13}C_{VAL}$-$\delta^{13}C_{PHE}$**	0.8	N/A	0.2	N/A
**$\delta^{13}C_{LEU}$-$\delta^{13}C_{PHE}$**	-2.2	N/A	-2.6	N/A

Supplementary References

1. Petersen, A. The archaeology of death and burial in the Islamic world. in *The Oxford Handbook of the Archaeology of Death and Burial* 241–258 (2013).
2. Halevi, L. *Muhammad's Grave: Death Rites and the Making of Islamic Society*. (Columbia University Press, 2007).
3. Sadan, J. On tombs and Holy Writ. Some methodological and lexicographical notes on burial concepts in Islamic fiqh (religious law), literature and practices. *Milest. Arts Cult. Egypt Tel Aviv Tel Aviv Univ.* 171–195 (2000).
4. Gleize, Y. *et al.* Early medieval Muslim graves in France: First archaeological, anthropological and palaeogenomic evidence. *PLoS ONE* **11**, e0148583 (2016).
5. Dury, G. *et al.* The Islamic cemetery at 33 Bartomeu Vicent Ramon, Ibiza: Investigating diet and mobility through light stable isotopes in bone collagen and tooth enamel. *Archaeol. Anthropol. Sci.* **11**, 3913–3930 (2019).
6. Avni, G. *The Byzantine-Islamic Transition in Palestine: An Archaeological Approach*. (Oxford University Press, 2014).
7. Herriott, C. Excavations at Ramla (Ta'avura Junction)-2011: Early Islamic graves and other features. *NGSBA Archaeol.* **2**, 131–139 (2013).
8. Avni, G. From standing stones to open mosques in the Negev Desert: The archaeology of religious transformation on the fringes. *East. Archaeol.* **70**, 124–138 (2007).
9. Moore, S. Burials and identities at historic period Çatal Höyük. *Herit. Turk.* **4**, 29 (2014).
10. Haddow, S. D. *et al.* Out of Range? Non-Normative Funerary Practices from the Neolithic to the Early Twentieth Century at Çatalhöyük, Turkey. in *The Odd, the Unusual, and the Strange: Bioarchaeological Explorations of Atypical Burials* 323–346 ({University of Florida Press, 2020).
11. Addison, E. Qastal 1998-2001. *ACOR Newsl.* **12**, 91–94 (2000).
12. Perry, M., Parker, K. & Montgomery, R. T. An early Islamic homicide at Qasr Hallabat, Jordan. *East. Archaeol.* **78**, 274–284 (2015).
13. McMillan, G. P. & Boone, J. L. Population history and the islamization of the Iberian Peninsula: Skeletal evidence from the Lower Alentejo of Portugal. *Curr. Anthropol.* **40**, 719–726 (1999).
14. del Campo, M. D. *et al.* An approach towards the effects of Muslim burial rites on skull preservation. Two cases from Hispanic Muslim cemeteries. *Anthropol. 1962-* **55**, 285–296 (2017).
15. Golding-Meir, N. Excavations at Ramla (Bialik interchange)-2010: Early Islamic graves. *NGSBA Archaeol.* **2**, 131–139 (2013).

16. Eshed, V. Anthropological report of the Ramla (Bialik interchange) Excavation. *NGSBA Archaeol.* **2**, 123–130 (2013).

17. Donner, F. M. *The early Islamic conquests*. (Princeton University Press, 2014).

18. Genequand, D. Umayyad Desert Palaces. *The Encyclopedia of Ancient History* vol. 1 2042–2044 (2013).

19. Le Strange, G. *Palestine under the Moslems: A Description of Syria and the Holy Land from AD 650 to 1500*. (Cosimo, Inc., 2010).

20. Guérin, A. Le village de Mseikeh et le Leja à la période islamique (VIIIe-XVe siècle). Archéologie du peuplement et histoire des territoires. in *Hauran III. L'habitat dans les campagnes de Syrie du Sud aux époques classique et médiévale* 261–273 (2008).

21. Guérin, A. Interprétation d'une register fiscal Ottoman: les territoires de la Syrie Méridonale en 1005/1597-97. *J. East. Stud.* **66**, 229–278 (2002).

22. Wright, W. *An account of Palmyra and Zenobia: with travels and adventures in Bashan and the desert*. (T. Nelson and sons, 1895).

23. Hütteroth, W.-D. & Abdulfattah, K. *Historical geography of Palestine, Transjordan and southern Syria in the late 16th century*. (Fränkische Geographische Ges., 1977, 1977).

24. Firro, K. *A History of the Druzes*. vol. 1 (Brill, 1991).

25. Porter, J. L. *Five years in Damascus, including an account of the history, topography and antiquities of that city: with travels and researches in Palmyra, Lebanon and the Hauran*. (John Murray, 1855).

26. Fowden, E. K. *The Barbarian Plain: Saint Sergius between Rome and Iran*. vol. 28 (Univ of California Press, 1999).

27. Guérin, A. Architecture villageoise et tribu nomade. Définition d'une peuplement dans la Lagâ à la période islamique (Syrie méridionale). *Berytus Archaeol. Stud.* 79–107 (1999).

28. Lancaster, W. & Lancaster, F. Graves and funerary monuments of the Ahl al-Ǧabal, Jordan. *Arab. Archaeol. Epigr.* **4**, 151–169 (1993).

29. Mustafa, M. H. & Abu Tayeh, S. N. Comments on Bedouin funeral rites in the writings of western travelers and explorers from the late 19th and early 20th centuries. *Mediterr. Archaeol. Archaeom.* **14**, 51–63 (2014).

30. Schaefer, J. Archaeological remains from the medieval Islamic occupation of the northwest Negev Desert. *Bull. Am. Sch. Orient. Res.* **274**, 33–60 (1989).

31. Zias, J. The cemeteries of Qumran and celibacy: confusion laid to rest? *Dead Sea Discov.* **7**, 220–253 (2000).

32. Moore, A. M., Hillman, G. C. & Legge, A. J. *Village on the Euphrates:From Foraging to Farming at Abu Hureya*. (Oxford Univesity Press, 2000).

33. Horowitz, L. & Kerbis, J. Hyenas at home. *Isr. - Land Nat.* **16**, 162–165 (1991).

34. Munt, H. *The Holy City of Medina. Sacred Space in Early Islamic Arabia*. (Cambridge University Press, 2014).
35. Burckhardt, J. L. *Notes on the Bedouins and Wahabys*. (H. Colburn and R. Bentley, 1830).
36. Cooper, P. The ruins that inspired pre-Islamic poets hold new meaning for today's Arabic writers. *The ancient poems that explain today* https://www.bbc.com/culture/article/20180820-the-6th-century-poems-making-a-comeback (2018).
37. Moore, S. Bones of evidence: the historic cemeteries of Çatalhöyük. *Herit. Turk.* **5**, 13–14 (2015).
38. Conrad, L. I. Tāʿūn and wabāʾ Conceptions of Plague and Pestilence in Early Islam. *J. Econ. Soc. Hist. OrientJournal Hist. Econ. Soc. Orient* **25**, 268–307 (1982).
39. Dols, M. W. Plague in early Islamic history. *J. Am. Orient. Soc.* **94**, 371–383 (1974).
40. Sartre, M. Bostra. Des origines à l'Islam. (1985).
41. Petersen, A. *The Medieval and Ottoman Hajj Route in Jordan; an Archaeological and Historical Study*. (Oxbow Books, 2012).
42. Haber, M. *et al.* A Transient Pulse of Genetic Admixture from the Crusaders in the Near East Identified from Ancient Genome Sequences. *Am. J. Hum. Genet.* **104**, 977–984 (2019).
43. Markus, B., Alshafee, I. & Birk, O. Deciphering the fine-structure of tribal admixture in the Bedouin population using genomic data. *Heredity* **112**, 182 (2014).
44. Almarri, M. A. *et al.* The Genomic History of the Middle East. *bioRxiv* 2020.10.18.342816 (2020) doi:10.1101/2020.10.18.342816.
45. Kivisild, T. *et al.* Ethiopian Mitochondrial DNA Heritage: Tracking Gene Flow Across and Around the Gate of Tears. *Am. J. Hum. Genet.* **75**, 752–770 (2004).
46. O'Leary, N. A. *et al.* Reference sequence (RefSeq) database at NCBI: current status, taxonomic expansion, and functional annotation. *Nucleic Acids Res.* **44**, D733-745 (2016).
47. Herbig, A. *et al.* MALT: Fast alignment and analysis of metagenomic DNA sequence data applied to the Tyrolean Iceman. *bioRxiv* 050559 (2016) doi:10.1101/050559.
48. Breitwieser, F. P., Baker, D. N. & Salzberg, S. L. KrakenUniq: confident and fast metagenomics classification using unique k-mer counts. *Genome Biol.* **19**, 198 (2018).
49. Langmead, B. & L. Salzberg, S. Fast gapped-read alignment with Bowtie 2 | Nature Methods. *Nat. Methods* **9**, 357 (2012).
50. Key, F. M., Posth, C., Krause, J., Herbig, A. & Bos, K. I. Mining Metagenomic Data Sets for Ancient DNA: Recommended Protocols for Authentication. *Trends Genet.* **33**, 508–520 (2017).
51. Hasan, M. J., Nizhu, L. N. & Rabbani, R. Bloodstream infection with pandrug-resistant Alcaligenes faecalis treated with double-dose of tigecycline. *IDCases* **18**, e00600 (2019).

52. Singla, N., Kaistha, N., Gulati, N. & Chander, J. Morganella morganii could be an important Intensive Care Unit pathogen. *Indian J. Crit. Care Med. Peer-Rev. Off. Publ. Indian Soc. Crit. Care Med.* **14**, 154–155 (2010).

53. Gupta, R. S., Lo, B. & Son, J. Phylogenomics and Comparative Genomic Studies Robustly Support Division of the Genus Mycobacterium into an Emended Genus Mycobacterium and Four Novel Genera. *Front. Microbiol.* **9**, 67 (2018).

54. Teebi, A. S. & Teebi, S. A. Genetic diversity among the Arabs. *Public Health Genomics* **8**, 21–26 (2005).

55. Haber, M. Isotopes Data from Ancient Lebanon. figshare. Dataset. (2020) doi:https://doi.org/10.6084/m9.figshare.12933293.v1.

56. Haber, M. *et al.* A genetic history of the Near East from an aDNA time course sampling eight points in the past 4,000 Years. *Am. J. Hum. Genet.* **107**, 149–157 (2020).

57. Sołtysiak, A. & Schutkowski, H. Continuity and change in subsistence at Tell Barri, NE Syria. *J. Archaeol. Sci. Rep.* **2**, 176–185 (2015).

58. Sandias, M. & Gundula, M. Diet and herding strategies in a changing environment: Stable isotope analysis of Bronze Age and Late Antique skeletal remains from Ya'amūn, Jordan. *J. Archaeol. Sci.* **63**, 24–32 (2015).

59. Al-Bashaireh, K., Al-Shorman, A., Rose, J., Jull, A. J. T. & Hodgins, G. Paleodiet Reconstruction of Human Remains from the Archaeological Site of Natfieh, Northern Jordan. *Radiocarbon* **52**, 645–652 (2010).

60. Gregoricka, L. A. & Sheridan, S. G. Ascetic or affluent? Byzantine diet at the monastic community of St. Stephen's, Jerusalem from stable carbon and nitrogen isotopes. *J. Anthropol. Archaeol.* **32**, 63–73 (2013).

61. Gregoricka, L. A. & Judd, M. A. Isotopic Evidence for Diet Among Historic Bedouin of Khirbat al-Mudayna, Jordan. *Int. J. Osteoarchaeol.* **26**, 705–715 (2016).
