# Supplementary material for: Bioarchaeological evidence of one of the earliest Islamic burials in the Levant
Source: Commun Biol. 2022 Jun 7;5:554. doi: 10.1038/s42003-022-03508-4 (PMC9174286; doi:10.1038/s42003-022-03508-4)
Supplement: Supplementary file 4 — Reporting Summary [file 42003_2022_3508_MOESM4_ESM.pdf]

nature research

Corresponding author(s): Torsten Günther, Cristina Valdiosera

Last updated by author(s): Mar 1, 2021

Reporting Summary

Nature Research wishes to improve the reproducibility of the work that we publish. This form provides structure for consistency and transparency in reporting. For further information on Nature Research policies, see our Editorial Policies and the Editorial Policy Checklist.

Statistics

For all statistical analyses, confirm that the following items are present in the figure legend, table legend, main text, or Methods section.

n/a	Confirmed	
☐	☒	The exact sample size (n) for each experimental group/condition, given as a discrete number and unit of measurement
☒	☐	A statement on whether measurements were taken from distinct samples or whether the same sample was measured repeatedly
☐	☒	The statistical test(s) used AND whether they are one- or two-sided<br>*Only common tests should be described solely by name; describe more complex techniques in the Methods section.*
☒	☐	A description of all covariates tested
☒	☐	A description of any assumptions or corrections, such as tests of normality and adjustment for multiple comparisons
☐	☒	A full description of the statistical parameters including central tendency (e.g. means) or other basic estimates (e.g. regression coefficient) AND variation (e.g. standard deviation) or associated estimates of uncertainty (e.g. confidence intervals)
☐	☒	For null hypothesis testing, the test statistic (e.g. F, t, r) with confidence intervals, effect sizes, degrees of freedom and P value noted<br>*Give P values as exact values whenever suitable.*
☒	☐	For Bayesian analysis, information on the choice of priors and Markov chain Monte Carlo settings
☒	☐	For hierarchical and complex designs, identification of the appropriate level for tests and full reporting of outcomes
☒	☐	Estimates of effect sizes (e.g. Cohen's d, Pearson's r), indicating how they were calculated

Our web collection on statistics for biologists contains articles on many of the points above.

Software and code

Policy information about availability of computer code

Data collection	No software was used for data collection.
Data analysis	All software packages used for data analysis are cited in the Materials and Methods section and are publicly available. Read mapping: Burrows-Wheeler Aligner (BWA, v0.7.15); Library merging: samtools version 1.5; mtDNA contamination estimates: contamMix (1.0-10). Autosomal contamination estimates: VerifyBAMID (1.1.3); X-chromosomal contamination estimates: ANGSD version 0.921; Mitochondrial Haplotype assignment: Web-based applications - HaploFind and HaploGrep; Calling SNPs on Y-chromosome of syr005: ISOGG database; Pruning SNPs in LD: PLINK version 1.9; PCA: smartpca (EIGENSOFT package); Model-based clustering: ADMIXTURE (1.3.0); visualization of clusters: Pong version 1.4.7; modelling ancestry: qpAdm (ADMIXTOOLS package v7.0.1); Outgroup f3 and D-statistics: Python script POPSTATS; Calling phenotypic variants: samtools version 1.5

For manuscripts utilizing custom algorithms or software that are central to the research but not yet described in published literature, software must be made available to editors and reviewers. We strongly encourage code deposition in a community repository (e.g. GitHub). See the Nature Research guidelines for submitting code & software for further information.

Data

Policy information about availability of data

All manuscripts must include a data availability statement. This statement should provide the following information, where applicable:

- Accession codes, unique identifiers, or web links for publicly available datasets
- A list of figures that have associated raw data
- A description of any restrictions on data availability

Generated sequence data will be available at the European Nucleotide Archive (ENA) under the accession number PRJEB38008.

Field-specific reporting

Please select the one below that is the best fit for your research. If you are not sure, read the appropriate sections before making your selection.

☒ Life sciences ☐ Behavioural & social sciences ☐ Ecological, evolutionary & environmental sciences

For a reference copy of the document with all sections, see nature.com/documents/nr-reporting-summary-flat.pdf

Life sciences study design

All studies must disclose on these points even when the disclosure is negative.

Sample size	Genomic and isitopic data from two individuals was analysed in this study.
Data exclusions	Reads shorter than 35 bp, showing more than 10% mismatch with the reference and/or a mapping quality score below 30 were discarded while merging data to prepare bamfiles of merged libraries. For population genetic analysis, minimum mapping and read qualities were 30 were used, transition sites were coded as missing to avoid post-mortem damage and pseudo-haploid representations were generated by randomly drawing one allele at each SNP site. For ADMIXTURE, 87098 out of 602366 SNPs in LD were removed prior to running the program. For calling phenotypic variants, only reads with base and mapping quality above 30 were used.
Replication	All filtering steps, datasets and parameters chosen are detailed in the Materials and Methods section.
Randomization	Not relevant to this study.
Blinding	N/A

Reporting for specific materials, systems and methods

We require information from authors about some types of materials, experimental systems and methods used in many studies. Here, indicate whether each material, system or method listed is relevant to your study. If you are not sure if a list item applies to your research, read the appropriate section before selecting a response.

Materials & experimental systems

n/a	Involved in the study	
☒	☐	Antibodies
☒	☐	Eukaryotic cell lines
☐	☒	Palaeontology and archaeology
☐	☒	Animals and other organisms
☒	☐	Human research participants
☒	☐	Clinical data
☒	☐	Dual use research of concern

Methods

n/a	Involved in the study	
☒	☐	ChIP-seq
☒	☐	Flow cytometry
☒	☐	MRI-based neuroimaging

Palaeontology and Archaeology

Specimen provenance	Two samples, syr005 and syr013, were excavated in Syria at the archaeological site of Tell Qarassa supported by the Spanish Ministry of Economy and Competitiveness (grant HAR2016-74999-P) and the Palarq Foundation (2010-2015). All appropriate permits for excavations and removal of samples were obtained by the Directorate- General of Antiquities and Museums from Syria.
Specimen deposition	All material was deposited at the Archaeological Museum of As-Suwayda (Syria).

Dating methods

Both samples were radiocarbon dated by AMS and calibrated using the Oxcal 4.3 program75, and the IntCal13 calibration curve.

☒ Tick this box to confirm that the raw and calibrated dates are available in the paper or in Supplementary Information.

Ethics oversight

All permits for site excavation and specimen sampling were obtained by the appropriate insititutions.

Note that full information on the approval of the study protocol must also be provided in the manuscript.

Animals and other organisms

Policy information about studies involving animals; ARRIVE guidelines recommended for reporting animal research

Laboratory animals	n/a
Wild animals	n/a
Field-collected samples	n/a
Ethics oversight	n/a

Note that full information on the approval of the study protocol must also be provided in the manuscript.
